# Alternative Splicing of *TaGS3* Differentially Regulates Grain Weight and Size in Bread Wheat

**DOI:** 10.3390/ijms222111692

**Published:** 2021-10-28

**Authors:** Xiaoli Ren, Liya Zhi, Lei Liu, Deyuan Meng, Qiannan Su, Aamana Batool, Jun Ji, Liqiang Song, Na Zhang, Lin Guo, Xigang Liu, Junming Li, Wei Zhang

**Affiliations:** 1 Center for Agricultural Resources Research, Institute of Genetics and Developmental Biology, The Innovative Academy of Seed Design, Chinese Academy of Sciences, Shijiazhuang 050022, China; renxiaoli2011@126.com (X.R.); xiaoya_19861028@163.com (L.Z.); mengdeyuan123@163.com (D.M.); SUQIANNAN1923@126.com (Q.S.); aamana.batool@yahoo.com (A.B.); jijun@sjziam.ac.cn (J.J.); lqsong@sjziam.ac.cn (L.S.); zhangna2nina@163.com (N.Z.); 2 The College of Life Science, University of Chinese Academy of Sciences, Beijing 100049, China; 3 School of Life Science, Huaiyin Normal University, Huaian 223300, China; leiliu_cell@163.com; 4 State Key Laboratory of Plant Cell and Chromosome Engineering, Chinese Academy of Sciences, Beijing 100101, China; 5 Ministry of Education Key Laboratory of Molecular and Cellular Biology, Hebei Collaboration Innovation Center for Cell Signaling, Hebei Key Laboratory of Molecular and Cellular Biology, College of Life Sciences, Hebei Normal University, Shijiazhuang 050024, China; linguomail@163.com (L.G.); xgliu@hebtu.edu.cn (X.L.); 6 College of Bioscience and Engineering, Hebei University of Economics and Business, Shijiazhuang 050061, China

**Keywords:** bread wheat, *TaGS3*, alternative splicing, grain weight, grain size

## Abstract

The heterotrimeric G-protein mediates growth and development by perceiving and transmitting signals in multiple organisms. Alternative splicing (AS), a vital process for regulating gene expression at the post-transcriptional level, plays a significant role in plant adaptation and evolution. Here, we identified five splicing variants of G_γ_ subunit gene *TaGS3* (*TaGS3.1* to *TaGS3.5*), which showed expression divergence during wheat polyploidization, and differential function in grain weight and size determination. *TaGS3.1* overexpression significantly reduced grain weight by 5.89% and grain length by 5.04%, while *TaGS3.2*–*3.4* overexpression did not significantly alter grain size compared to wild type. Overexpressing *TaGS3.5* significantly increased the grain weight by 5.70% and grain length by 4.30%. Biochemical assays revealed that *TaGS3* isoforms (TaGS3.1–3.4) with an intact OSR domain interact with WGB1 to form active G_βγ_ heterodimers that further interact with WGA1 to form inactive G_αβγ_ heterotrimers. Truncated isoforms *TaGS3.2–3.4* , which lack the C-terminal Cys-rich region but have enhanced binding affinity to WGB1, antagonistically compete with *TaGS3.1* to bind WGB1, while *TaGS3.5* with an incomplete OSR domain does not interact with WGB1. Taking these observations together, we proposed that *TaGS3* differentially regulates grain size via AS, providing a strategy by which the grain size is fine-tuned and regulated at the post-transcriptional level.

## 1. Introduction

Bread wheat (*Triticum aestivum* L.) is a global staple crop. High yield, as the prime breeding target in cereals, is determined by the panicles per plant, grain number per spike, and grain weight. Grain weight is the major determinant of yield potential, and it is largely dependent on grain size. Thus, grain size has long been the critical target of selection in wheat breeding [1].

Genetic and molecular analyses have identified numerous genes involved in multiple signaling pathways that regulate grain size [2]. One of the most important pathways is the G-protein signaling pathway. Heterotrimeric G-proteins, comprising G_α_, G_β_, and G_γ_ subunits, are the molecular switches that transmit signals from transmembrane receptors to downstream target proteins. G_γ_ binds tightly to G_β_, forming a functional G_βγ_ unit that can only be dissociated under denaturing conditions [3]. The structural diversity of G_γ_ subunits solely provides functional selectivity to the heterotrimer [4]. G_γ_ subunits are divided into three types according to their C-terminal structures, of which the atypical G_γ_ subunit both contains a plant-specific organ size regulation (OSR) domain in the N terminus, and possesses a C-terminal Cys-rich region [5].

One noncanonical G_γ_ subunit (AGG3) is a positive regulator of seed size in Arabidopsis [6] because the null *agg3* mutant produces smaller seeds and organs than those of wild type (WT), but *AGG3* overexpression lines generate larger seeds and organs. OsGS3, a G_γ_ subunit in rice, acts as a negative regulator of grain weight and length [7]. In de novo domestication, *GS3* sgRNA targeting significantly increased grain length in PPR1 mutants of wild allotetraploid rice [8]. A mutant with an allelic variation resulting in the truncated OSR domain of OsGS3 produces long grains [9], whereas the OsGS3 mutant with deletion of the C-terminal Cys-rich region produces very short grains [10], indicating the divergent functions of the N-terminal OSR domain and the C-terminal Cys-rich region of OsGS3 in regulating rice grain size. In wheat, three *TaGS3* homeologous genes were identified on chromosomes 7A, 4A and 7D (designed as *TaGS3-7A*, *TaGS3-4A* and *TaGS3-7D*), which negatively regulate grain weight and size [11]. Although multiple haplotypes in *TaGS3* homeologs have been identified, the underlying molecular mechanisms determining the existence of these multiple haplotypes are still unclear.

Alternative splicing (AS) is a key post-transcriptional regulatory mechanism for expanding proteomic diversity and functional complexity [12], even generating trait diversity [13]. AS mainly results in generation of an alternative 3′ or 5′ splicing site (Alt 3′ ss and Alt 5′ ss, respectively),) intron retention (IR), and exon skipping (ES) [13,14]. AS can produce truncated proteins, disrupting key domains or abolishing interaction with other proteins and thereby preventing the formation of functional protein complexes [15,16]. Moreover, some splicing variants can compete with a constitutive variant and interfere with its function in a dominant negative manner [17,18]. In our previous study [11], we observed the expression of multiple similar transcripts during *TaGS3* cloning. The differences between the *TaGS3* transcripts and their biological functions were unclear.

AS participates in the regulation of a series of grain-related traits. A sequence duplication at exon 2 of *Psy-A1* creates a new splice site and causes AS with an activated cryptic exon, resulting in four splicing variants. Only the constitutive splicing variant produces an enzymatically active protein, and its mRNA abundance is reduced by titration with the other splicing variants, which is argued to result in a reduction in PSY and thus carotenoid content in wheat [19]. Two mRNA variants are found for *Delay of Germination1* (*DOG1*), *lgDOG1* and *shDOG1*. *shDOG1* is translated and functional, in contrast to *lgDOG1*, promoting seed dormancy in Arabidopsis [20]. AS of several genes was reported to affect rice grain size. *OsLG3b* encodes MADS-box transcription factor1 (*OsMADS1*). Its six SNP variations lead to AS, introducing a premature termination codon (PTC), and resulting in the truncation of 32 amino acid residues that are positively associated with grain length [21]. *OrMKK3* undergoes AS and produces splicing variants. The overexpression of four of the five *OrMKK3* splicing variants results in reduced grain length and width [22]. Nevertheless, whether and how these splicing variants interact with each other to modulate agronomic traits remain unknown.

Bread wheat has a huge and complex genome with a number of genes playing complex roles [23]. The genome-wide profiling of AS revealed a complex AS landscape in wheat, showing that approximately 22.1% of genes exhibit AS [24]. AS dynamics and evolutionary divergence during embryogenesis in wheat species indicated that AS provides opportunities for transcriptional and proteomic plasticity, and the potential for generating trait diversity [12]. Some reports have illustrated that AS events in wheat are substantially related to grain biochemical traits such as phytoene synthase activity [19], polyphenol oxidase activity [25], and starch synthase activity [26]. AS is also involved in disease resistance [27] and abiotic stresses tolerance [28,29,30]. Studies related to AS on wheat grain traits are mainly focused on grain quality. AS events and their biological functions in wheat genes related to grain weight and size remain poorly understood.

Here, we report that *TaGS3* undergoes AS and produces five splicing variants, corresponding to the constitutive *TaGS3.1*, the truncated *TaGS3.2–3.4* lacking the C-terminal Cys-rich region, and the truncated *TaGS3.5* that contains the disrupted OSR domain. Evolutionary analysis showed that AS of *GS3* did not emerge during the process of wheat polyploidization, but pre-existed in the ancestor species of *Triticeae* crops. Given protein–protein interactions between *TaGS3* isoforms and WGB1 (G_β_), we biochemically analyzed their nature and found that *TaGS3.2*, *TaGS3.3*, and *TaGS3.4* interfere with *TaGS3.1* function by competitively forming functional G_βγ_ heterodimers, and that *TaGS3.5* is unable to form a G_βγ_ heterodimer with WGB1. Correspondingly, overexpression of the five *TaGS3* splicing variants in bread wheat exhibited divergent effects on grain development. In particular, *TaGS3.5* overexpression produced significantly larger and heavier grains. Taken together, these observations support the proposal that the TaGS3-mediated signal transduction pathway regulates grain weight and size via AS, providing a strategy by which the regulation of grain size is fine-tuned and balanced at the post-transcriptional level.

## 2. Results

### 2.1. AS Occurrence of TaGS3 in Wheat

**Figure 1:**
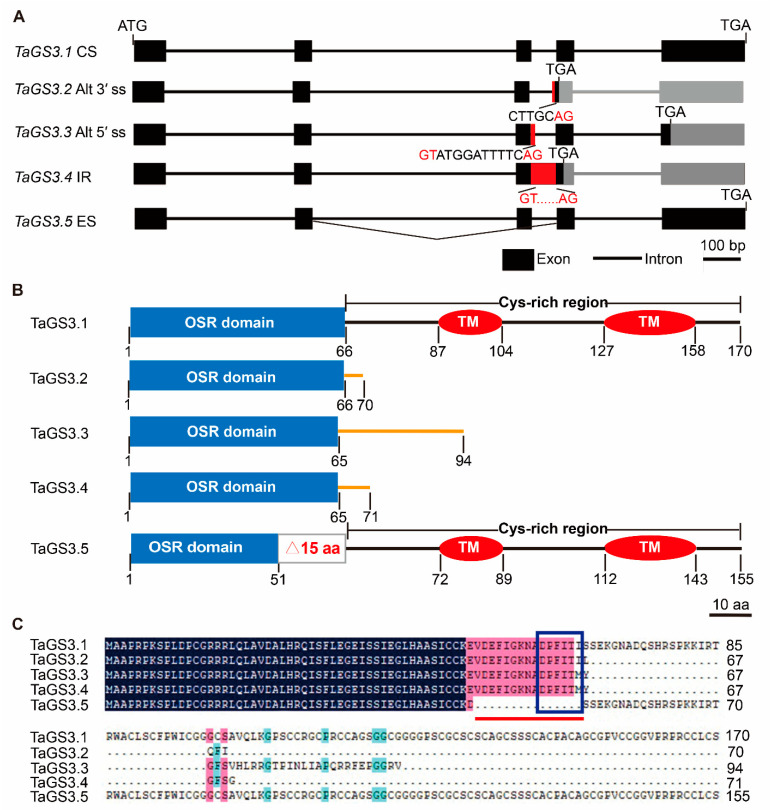
*TaGS3* is subject to AS.(**A**) Schematic representation of gene structures of *TaGS3* splicing variants. CS, constitutive splicing; Alt 3′ ss, alternative 3′ splice site; Alt 55′ ss, alternative 5′ splice site; IR, intron retention; ES, exon skipping. Gene exons and introns are shown by black boxes and lines, respectively. *TaGS3* splicing variants are distinguished by red boxes (intron 3 retention section) and polyline (exon 3 skipping). GT…AG represents the canonical splicing site. Bar = 100 bp. (**B**) The structural diagrammatic sketch of the five *TaGS3* isoforms. Bar = 10 aa. OSR, organ size regulation domain; TM, putative transmembrane domain. The orange lines indicate the C-terminal non-Cys-rich region. (**C**) Protein sequence alignment of the *TaGS3* isoforms. The 15 missing amino acid residues of *TaGS3.5* are highlighted by red line. The DPFITI motif is marked by a blue quadrilateral.

A previous study indicated that *TaGS3* negatively regulates grain weight and size in wheat [10]. During *TaGS3* cloning, we identified several distinct CDSs of *TaGS3*. To decipher the identity of the transcripts, we sequenced the amplicons and confirmed the presence of five unique *TaGS3* transcripts (Figure S1). Except for the constitutive variant *TaGS3.1*, four splicing variants arise from Alt 3′ ss, Alt 5′ ss, IR, and ES, leading to the production of *TaGS3.2*, *TaGS3.3*, *TaGS3.4*, and *TaGS3.5*, respectively (Figure 1A).

*TaGS3.1* contains five exons and four introns, and is predicted to encode the full length γ-subunit protein of 170 amino acid residues, as previously reported [11]. Sequence analysis revealed that AS results in *TaGS3.2*, *TaGS3.3*, and *TaGS3.4* with varied degrees of retention in the third intron, with *TaGS3.2* harboring seven nucleotides (CTTGCAC) at the 3′-end of intron 3, *TaGS3.3* harboring 14 nucleotides (GTATGGATTTTCAG) at the 5′-end of intron 3, and *TaGS3.4* harboring the full length of intron 3 (82 bp). As a result, *TaGS3.2*, *TaGS3.3*, and *TaGS3.4* are all frame-shifted, with introduction of a PTC at the 213th nucleotide site of exon 4, 285th nucleotide site of exon 5, and 216th nucleotide site of exon 4, respectively. For *TaGS3.5*, AS has resulted in skipping the third exon (Figure 1A and Figure S2).

Homology comparison shows 88.36% and 42.92% similarities in the N terminus and C terminus of *GS3* in *T*. *aestivum*, *H. vulgare*, *S. italica*, *S. bicolor* (L.) *Moench*, *Z*. *mays*, *P. hallii*, *O*. *sativa*, and *B. distachyon* (Figure S3), indicating the high degree of conservation in the N terminus of *GS3* homologs. The C-terminal Cys-rich region of *GS3*, however, is mostly divergent in length and composition in *Poaceae* species. Rice OsGS3 possesses a conserved OSR domain (7–72 aa), sharing high similarity with the N-terminal regions of G_γ_ in a number of species, ranging from angiosperm to gymnosperm [10]. Alignment analysis of *TaGS3.1* and OsGS3 revealed the presence of conserved domains in TaGS3.1: one OSR domain (1–66 aa), two transmembrane domains (87–104 and 127–158 aa) and one Cys-rich region (67–170 aa) (Figure 1B and Figure S4A). The intron retention of *TaGS3.2*, *TaGS3.3*, and *TaGS3.4* results in the 70, 94, and 71 aa truncation of TaGS3, respectively, leading to deletion of the C-terminal Cys-rich region containing two predicted transmembrane domains (Figure S4B–D). In addition, *TaGS3.2* contains an intact OSR domain (1–66 aa), and the OSR domains in *TaGS3.3* and *TaGS3.4* show 1 aa difference (Figure 1B). The skipping of the third exon (45 bp) of *TaGS3.5* does not lead to a frame shift, and the predicted encoded peptide is 155 amino acid residues. This truncated protein lacks 15 amino acid residues, including the DPFITI motif at the 52nd amino acid residue site in the OSR domain (Figure 1C). Therefore, *TaGS3.5* is predicted to contain one incomplete OSR domain (1–51 aa) and two complete predicted transmembrane domains (72–89 aa, 112–143 aa) (https://embnet.vital-it.ch/software/TMPRED_form.html accessed on 15 August 2021) (Figure 1B and Figure S4E).

### 2.2. GS3 Splicing Variants in Wheat Species

**Figure 2:**
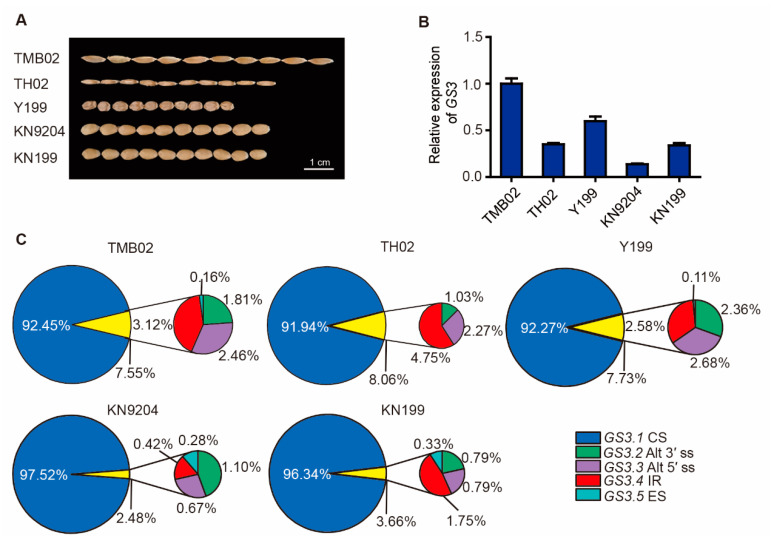
The occurrence frequency of *GS3* splicing variants in diploid wheat progenitors and hexaploid wheat. (**A**) Phenotypic comparison of grain traits among the indicated diploid wheat progenitors and hexaploid wheat cultivars. Bar = 1 cm. (**B**) Relative expression of *GS3* in mature seeds of the indicated diploid wheat progenitors and hexaploid wheat cultivars. Normalized expression values of *GS3* splicing variants relative to *GAPDH* were given as mean ± SEM from three replicates. (**C**) The occurrence frequency of *GS3* splicing variants in developing grains of the indicated diploid wheat progenitors and hexaploid wheat cultivars. The *GS3* splicing variants and AS types are represented by different colors (dark blue, green, purple, red, and light blue stand for *GS3.1* CS, *GS3.2* Alt 3′ ss, *GS3.3* Alt 5′ ss, *GS3.4* IR, and *GS3.5* ES, respectively).

To determine whether AS patterns changed during wheat polyploidization, we examined three diploid wheat progenitor species, namely, TMB02 (*T*. *boeoticum*, AA), TH02 (*Ae*. *sharonensis*, SS), and Y199 (*Ae*. *tauschii*, DD), and two *T*. *aestivum* cv. KN9204 and KN199, which showed divergent grain morphological variations and relative *GS3* expression levels (Figure 2A,B). Analyses on the occurrence frequency of the five *GS3* splicing variants in developing grains showed that *GS3.1* is the major splicing variant, representing 92.45%, 91.94%, and 92.27% in diploid TMB02, TH02, and Y199 and 97.52% and 96.34% in hexaploid KN9204 and KN199, respectively. The total ratio of the four other splicing variants accounted for 7.55%, 8.06%, 7.73%, 2.48%, and 3.66% in TMB02, TH02, Y199, KN9204, and KN199, respectively (Figure 2C).

Except for constitutive splicing variant *GS3.1*, the distributions of the four other *GS3* splicing variants were analyzed in diploid and hexaploid species. *GS3.4* is the most abundant splicing variant in TMB02 and TH02, followed by *GS3.3*, *GS3.2*, and *GS3.5*. There is no obvious difference in the proportion of *GS3.2*, *GS3.3*, and *GS3.4* in Y199, accounting for 2.36%, 2.68%, and 2.57%, respectively. In KN9204, *GS3.2* is the most abundant splicing variant, followed by *GS3.3*, *GS3.4* and *GS3.5*. In KN199, the distribution of *GS3* splicing variants is similar to that in TMB02 and TH02, with *GS3.4* being the most abundant splicing variant (Figure 2C). Taken together, *GS3.1*, *GS3.2*, *GS3.3*, and *GS3.4* variants are conserved in diploid species, while *GS3.5* shows divergent patterns, with a small portion of *GS3.5* transcript identified in TMB02 (0.16%) and Y199 (0.11%) but not in TH02. Moreover, *TaGS3* exhibited different splicing patterns among the triplex in hexaploid wheat. *GS3.1*, *GS3.3*, and *GS3.4* variants were all examined in the *TaGS3* triplex; *GS3.2* was only identified from *TaGS3-4A* and *TaGS3-7D*, which is attributed to the absence of one canonical splice junction (AG) in the third intron of *TaGS3-7A* compared with those of *TaGS3-4A* and *TaGS3-7D* (Figure S5). *GS3.5* was identified from *TaGS3-7A* and *TaGS3-4A* in KN9204, but from *TaGS3-4A* and *TaGS3-7D* in KN199 (Table S1).

### 2.3. Expression Patterns and Subcellular Localization of TaGS3 Splicing Variants

**Figure 3:**
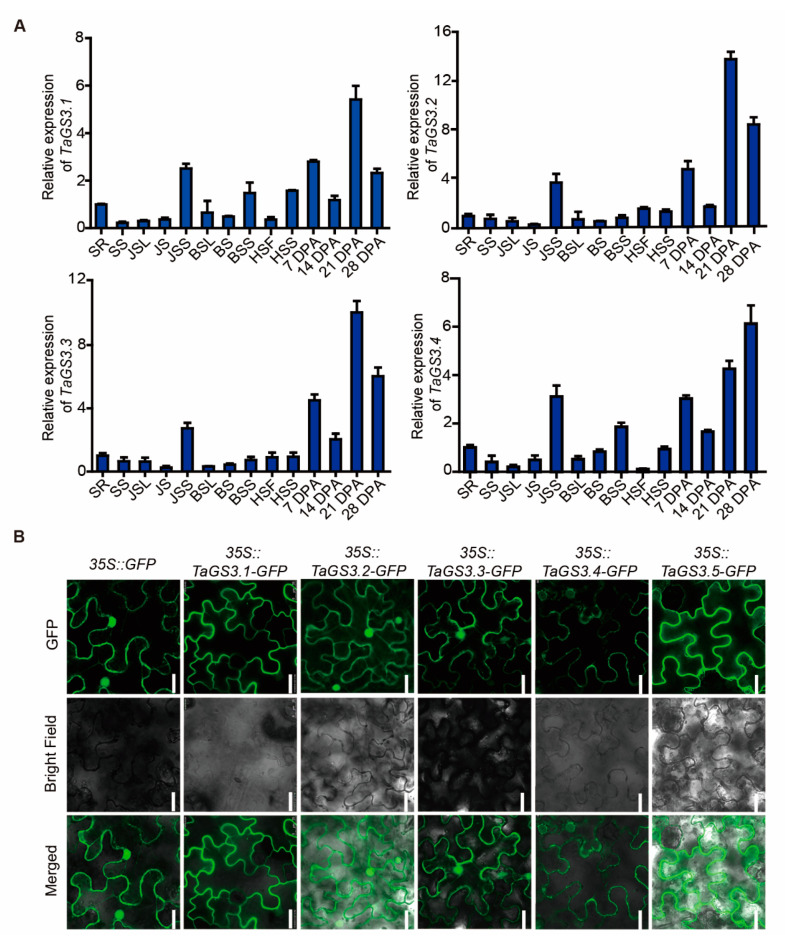
The expression patterns and subcellular localization of *TaGS3* splicing variants. (**A**) Expression analyses of the *TaGS3* splicing variants. SR, seedling root; SS, seedling shoot; JSL, leaves at jointing stage; JS, stems at jointing stage; JSS, spikes at jointing stage; BSL, leaves at booting stage; BS, stems at booting stage; BSS, spikes at booting stage; HSF, flag leaves at heading stage; HSS, spikes at heading stage; 7 DPA-28 DPA, grains at 7–28 days post-anthesis, respectively. Normalized expression values of *TaGS3* splicing variants relative to *GAPDH* were given as mean ± SEM from three replicates. (**B**) Subcellular localization of the *TaGS3* isoforms. *GFP* and the five *TaGS3* splicing variants fused with *GFP* under the control of the *CaMV 35S* promoter were transiently expressed in epidermal cells of *N. benthamiana*. Bar = 25 μm.

The temporal and spatial expression patterns of the five splicing variants of *TaGS3* were investigated in KN9204 through qRT-PCR using cDNA-specific primers. *TaGS3.1*, *TaGS3.2*, *TaGS3.3*, and *TaGS3.4* were ubiquitously expressed with similar patterns in various wheat tissues at different developmental stages, showing higher expression levels in young spikes and developing grains than in the roots, stems, and leaves. Nevertheless, there were notable differences in expression abundances among the 5 variants; the maximum expression levels of *TaGS3.1*, *TaGS3.2*, and *TaGS3.3* were observed in the 21 DPA (day post-anthesis) grains, *TaGS3.4* expression level peaked in the 28 DPA grains (Figure 3A), but *TaGS3.5* expression was undetectable in all assayed tissues by qRT-PCR.

AS frequently results in alterations in protein structures and subcellular locations [31]. To determine the subcellular localization of *TaGS3* isoforms, we expressed the five *TaGS3* splicing variants as fusions with GFP under the control of the *CaMV 35S* promoter in *N. benthamiana*. The localization of *TaGS3.1*-GFP and *TaGS3.5*-GFP fusion protein fluorescence was only observed in the plasma membrane, while *TaGS3.2*-GFP, *TaGS3.3*-GFP, and *TaGS3.4*-GFP fusion protein fluorescence was observed in both the plasma membrane and the nuclei (Figure 3B). Consistent with these results, *Arabidopsis* AGG3 was present in the plasma membrane, but AGG3^△108–125^, in which the predicted transmembrane domain is deleted, was observed in both the plasma membrane and the nucleus [6].

### 2.4. Overexpression of TaGS3 Splicing Variants Confers Different Effects on Wheat Grain Weight and Size

**Figure 4:**
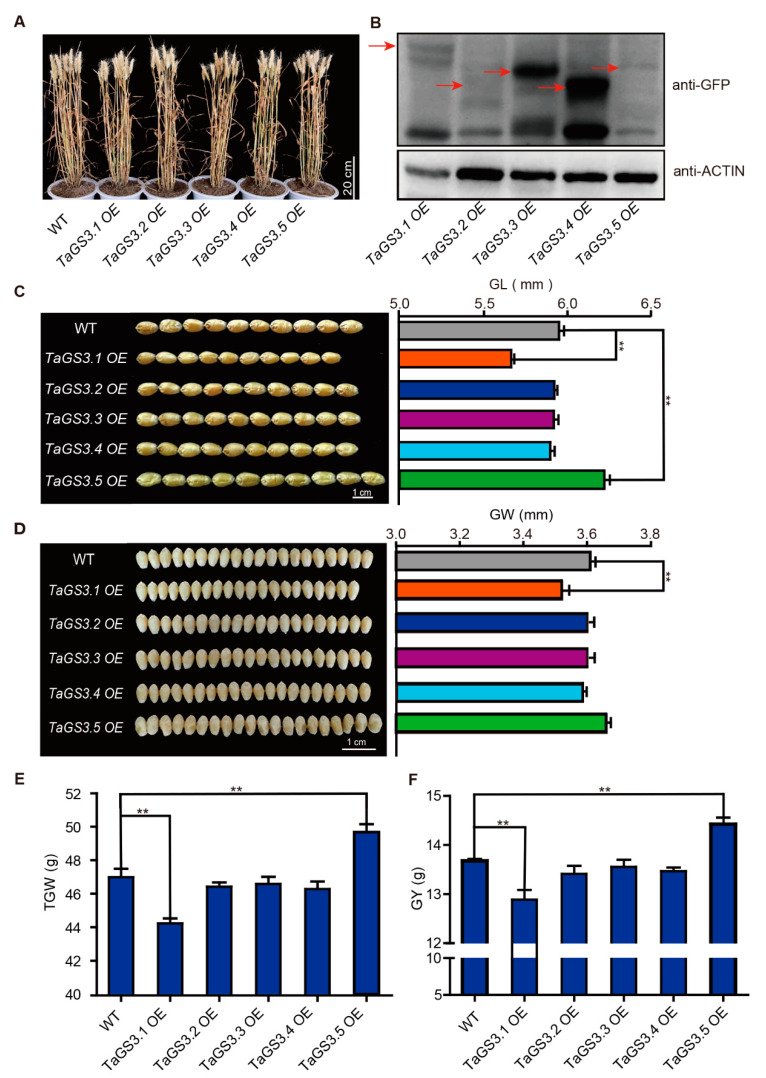
Phenotypic comparison of agronomic attributes among the overexpression lines of *TaGS3* splicing variants and WT under field conditions. (**A**) Overview of the whole plants of *TaGS3* splicing variants overexpression lines and WT. Bar = 20 cm. (**B**) Immunoblot analysis of the T_3_ transgenic plants of *TaGS3* splicing variants. Total protein was extracted from the leaves of 3-day-old seedlings for Western blotting. (**C**) Comparison of the grain length among the tested genotypes. Bar = 1 cm. (**D**) Comparison of the grain width among the tested genotypes. Bar = 1 cm. (**E**) Thousand-grain weight (TGW) and (**F**) grain yield per plant (GY) of the tested genotypes. ** *p* < 0.01 (Tukey test) indicates significant differences between the overexpression lines and WT. Data are given as mean ± SEM (n = 10).

To study the genetic effects of the five *TaGS3* splicing variants in bread wheat, we generated *TaGS3.1*, *TaGS3.2*, *TaGS3.3*, *TaGS3.4*, and *TaGS3.5* transgenic lines in *T. aestivum* cv. KN199 (Figure 4A). Fold increase in the expression abundances of *TaGS3* variants was examined in T_3_ homozygous overexpression lines (Figure S6). To investigate the relative abundance of the specific proteins, we tested the transgenic plants expressing GFP tagged-fusion of *TaGS3.1*, *TaGS3.2*, *TaGS3.3*, *TaGS3.4*, and *TaGS3.5* in KN199 driven by the ubiquitin promoter. Western blotting analysis showed that *TaGS3.1*-GFP, *TaGS3.2*-GFP, *TaGS3.3*-GFP, *TaGS3.4*-GFP, and *TaGS3.5*-GFP were all overexpressed in the transgenic bread wheat (Figure 4B).

The stability of *TaGS3* isoforms in the corresponding overexpression lines was assayed. Immunoblot analysis revealed trace expression of *TaGS3.2*-GFP that was barely detected, and the abundance of *TaGS3.3*-GFP and *TaGS3.4*-GFP was significantly reduced under the treatment of cycloheximide (CHX), a protein synthesis inhibitor, whereas the accumulation of *TaGS3.1*-GFP and *TaGS3.5*-GFP only changed slightly. The accumulation of all *TaGS3.2*-GFP, *TaGS3.3*-GFP, and *TaGS3.4*-GFP but not *TaGS3.1*-GFP and *TaGS3.5*-GFP increased under treatment with the proteasome inhibitor MG132 (Figure S7). These results indicate that *TaGS3.1* and *TaGS3.5* are more stable than *TaGS3.2–3.4* , implying the strong influence of the C-terminal Cys-rich region on TaGS3′s stability.

The agronomic attributes of *TaGS3.1*–*3.5* overexpression lines were examined in field studies conducted in 2019. No significant differences were found between *TaGS3.1*–*3.5* overexpression lines and WT in terms of plant height (PH), spike number per plant (SN), spike length (SL), total spikelets per spike (TS), sterile spikelets per spike (SS), and grain number per spike (GN) (Figure 4A; Table S2). However, significant differences were detected for each *TaGS3.1*–*3.5* overexpression line compared with WT in grain traits such as grain length (GL), grain width (GW), thousand-grain weight (TGW), and grain yield per plant (GY) (Figure 4 C–F). Compared to WT, *TaGS3.1* overexpression lines exhibited significantly smaller and lighter grains, with a reduction of 5.04% (0.30 ± 0.04 mm) in GL, 2.49% (0.08 ± 0.02 mm) in GW, 5.89% (2.77 ± 0.54 g) in TGW, and 5.77% (0.79 ± 0.19 g) in GY. There were no significant variations in GL, GW, TGW, and GY between *TaGS3.2*–*3.4* overexpression lines and WT, but a 0.39–0.95% decrease in GL, 0.80–1.52% decrease in TGW and 0.92–1.91% decrease in GY between *TaGS3.2–3.4* overexpression lines and WT. Notably, *TaGS3.5* overexpression lines produced significantly larger and heavier grains, with an increase of 4.39% (0.26 ± 0.03 mm) in GL, 1.39% (0.05 ± 0.02 mm) in GW, 5.70% (2.68 ± 0.65 g) in TGW, and 5.41% (0.74 ± 0.19 g) in GY (Figure 4C–F). During three growing seasons, all *TaGS3.1*–*3.5* overexpression lines displayed a recurring phenotype, similar to the results shown above (Table S2). These results suggested that *TaGS3.1* and *TaGS3.5* make opposing contributions to grain weight and grain size.

### 2.5. TaGS3.1, TaGS3.2, TaGS3.3, and TaGS3.4 Physically Interact with WGB1

**Figure 5:**
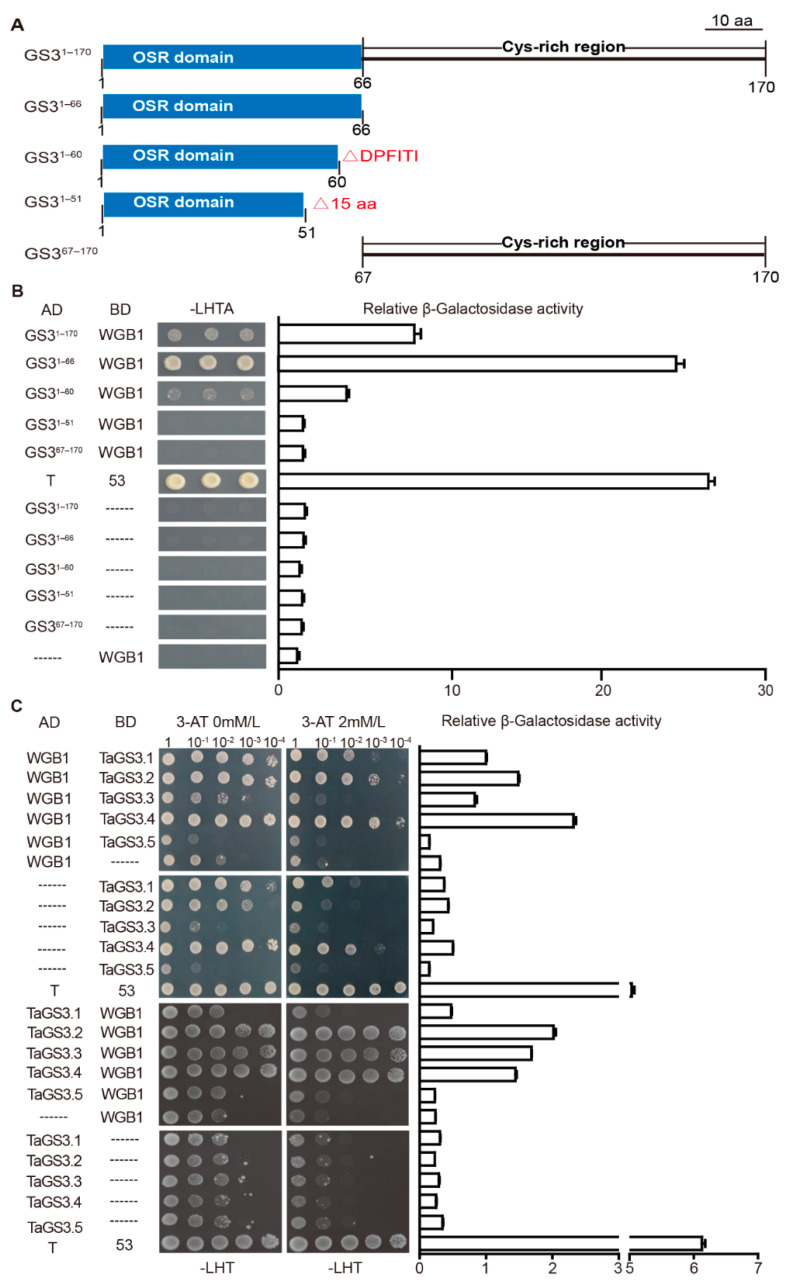
*TaGS3* interacts with WGB1 through the OSR domain, rather than the C-terminal Cys-rich region. (**A**) Schematic structure of protein versions with specific deleted domains. (**B**) Yeast two-hybrid assay showing the interactions of full-length *GS3*^1–170^ and the four truncated proteins (GS3^1–66^, *GS3*^1–60^, *GS3*^1–51^, and *GS3*^67–170^) with WGB1. AD, GAL4 activation domain; BD, GAL4 DNA binding domain; AD and BD represent empty pGADT7 and pGBKT7 vectors, respectively. AD-T/BD-53, positive control. -LHTA, selective medium lacking Leu, His, Trp and Ade. Quantitative analyses of interactions by β-galactosidase assay were shown (on the right) as mean ± SEM (n = 3). (**C**) Yeast two-hybrid assay showing the interactions of *TaGS3* isoforms with WGB1. -LHT, selective medium lacking Leu, His and Trp. The yeast strains were serially diluted (OD_600_, 10^−1^–10^−4^) before spotting on selection medium-synthetic dropout interaction medium -LHT. The specificity of the stringency of the assay was tested by adding 3-aminotriazole (3-AT, 0 mM/L and 2 mM/L, respectively). Quantitative analyses of interactions by β-galactosidase assay were shown (on the right) as mean ± SEM (n = 3).

The G protein consists of G_α_, G_β_ and G_γ_ subunits, where G_α_ and G_β_ play fundamental roles in the transduction of G protein-mediated growth signals, and G_β_-mediated signal transduction requires G_γ_ for formation of active G_βγ_ heterodimer [32,33]. To determine which section of *TaGS3* is essential for G_βγ_ dimer formation, we generated five *TaGS3* isoforms: the full-length *GS3*^1–170^ and the truncated *GS3*^1–66^, *GS3*^1–60^, *GS3*^1–51^, and *GS3*^67–170^. Different from *GS3*^1–170^, the *GS3*^1–66^, *GS3*^1–60^, and *GS3*^1–51^ isoforms were all devoid of the C-terminal Cys-rich region. *GS3*^1–66^ contains an intact OSR domain, while *GS3*^1–60^ contains an incomplete OSR domain lacking the six amino acid residues of the highly conserved DPFITI motif, and *GS3*^1–51^ contains an incomplete OSR domain that lacks 15 amino acid residues including the conserved DPFITI motif. By contrast, *GS3*^67–170^ only comprises the C-terminal Cys-rich region without the N-terminal OSR domain (Figure 5A). Yeast two-hybrid and β-galactosidase assays demonstrated that *GS3*^1–170^, *GS3*^1–66^, and *GS3*^1–60^ interact with WGB1, in which *GS3*^1–66^ exhibits stronger affinity to WGB1 compared to *GS3*^1−170^, while *GS3*^1–60^ shows weak affinity to WGB1, indicating that both the conserved DPFITI motif and the deletion of the C-terminal Cys-rich region are required for the enhancement of *GS3* binding to WGB1. *GS3*^1–51^ and *GS3*^67−170^, on the other hand, did not interact with WGB1 (Figure 5B). These results suggest that *GS3* interacts with WGB1 through the OSR domain rather than the C-terminal Cys-rich region, and the section of the 15 amino acid residues in the OSR domain is essential for the binding of *GS3* to WGB1.

**Figure 6:**
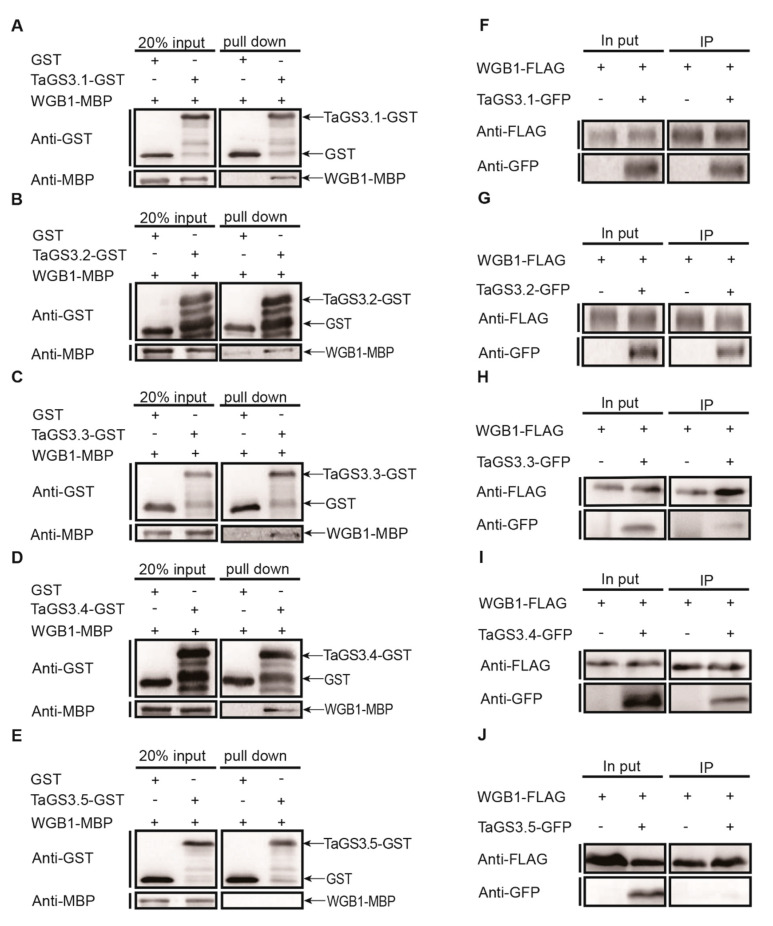
Interaction analyses of *TaGS3* isoforms with WGB1. (**A**–**E**) In vitro GST pull-down assays showing the interactions between *TaGS3* isoforms and WGB1. Recombinant maltose binding protein WGB1-MBP and five*TaGS3* isoforms-GST were used. (**F**–**J**) In vivo Co-IP assays showing the interactions between *TaGS3* isoforms and WGB1. FLAG-tagged WGB1 was co-expressed with GFP-tagged *TaGS3* isoforms in *N. benthamiana* leaves.

Subsequently, we addressed whether the five *TaGS3* isoforms encoded by *TaGS3* splicing variants form a heterodimeric complex with WGB1. Here, the amino acid sequence of *TaGS3.1* was the same as *GS3*^1–170^. Yeast two-hybrid and β-galactosidase assays confirmed that *TaGS3.2*, *TaGS3.3*, and *TaGS3.4* bind more tightly to WGB1 than TaGS3.1. *TaGS3.5*, lacking the 15 amino acid residue section in the OSR domain, does not bind to WGB1 (Figure 5). These results consistently support the necessity of the 15 amino acid residues in the OSR domain for the TaGS3-WGB1 interaction and the enhanced binding to WGB1 by *TaGS3* when the C-terminal Cys-rich region is deleted. Furthermore, these results of the yeast two-hybrid assay were confirmed by pull-down and coimmunoprecipitation (Co-IP) assays, where WGB1 interacted with *TaGS3.1*, *TaGS3.2*, *TaGS3.3*, and *TaGS3.4* instead of *TaGS3.5* in vitro (Figure 6A–J).

### 2.6. Competitive Interactions of TaGS3 Isoforms with WGB1

**Figure 7:**
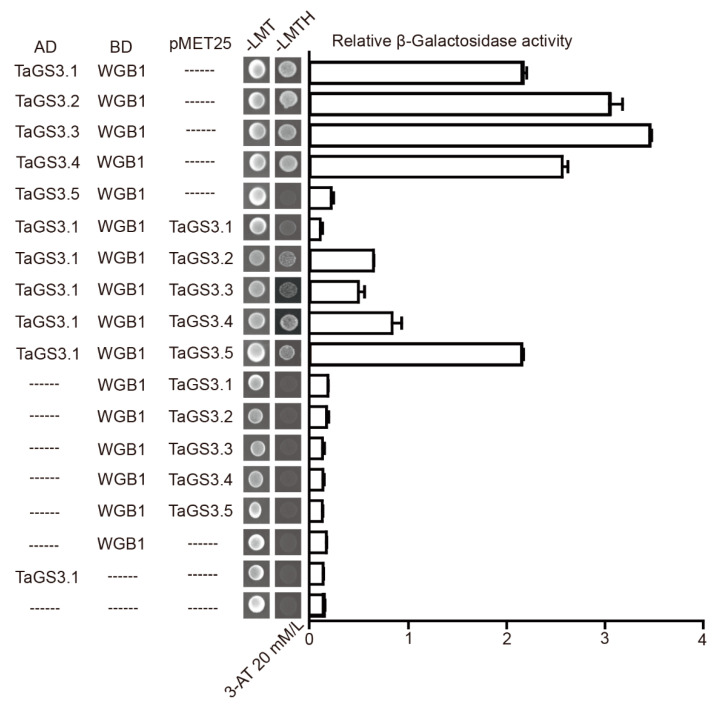
Competition test of the interactions between *TaGS3* isoforms and WGB1. Yeast three-hybrid assay showing the competition between *TaGS3.1* and the other four truncated *TaGS3* isoforms to interact with WGB1 by using fusions with AD (AD-*TaGS3* isoforms) and BD (BD-WGB1/BD-WGB1-GS3 isoform) (on the left). AD and BD represent empty pGADT7 and pBridge vectors, respectively. Empty vector was used as the negative control. -LMT, selective medium lacking Leu, Met, and Trp; -LMTH, selective medium lacking Leu, Met, Trp, and His. The specificity of the stringency of the assay was tested by adding 3-aminotriazole (3-AT, 20 mM/L). Quantitative analyses of interactions by β-galactosidase assay were shown (on the right) as mean ± SEM (n = 3).

In a nonactivated state, the G_α_ subunit maintains its GDP-bound state, forming an inactive heterotrimer with the G_βγ_ dimer [34]. To test whether *TaGS3* isoforms form a heterotrimer with WGB1 and WGA1, we performed yeast three-hybrid assay and found that *TaGS3.1*, *TaGS3.2*, *TaGS3.3*, and *TaGS3.4*, but not *TaGS3.5*, coupled with WGB1 to interact with WGA1 (Figure S8). In addition, yeast three-hybrid assay revealed competition between *TaGS3.2–3.4* and *TaGS3.1* in interaction with WGB1, where TaGS3.2–3.3 expression strongly disrupted the interaction of *TaGS3.1* and WGB1, but *TaGS3.4* expression slightly influenced interaction of *TaGS3.1* and WGB1 (Figure 7). These results suggested competitive interactions between *TaGS3.2–3.4* and *TaGS3.1*, providing an explanation for the functional existence and effects of multiple *TaGS3* splicing variants for allowing fine-tuning of the regulation of wheat grain size.

## 3. Discussion

### 3.1. Conservation and Significance of GS3 AS in Gramineae

Recent large-scale genomics studies, especially genome-wide analysis of gene expression profiling, revealed that 30% to 61% of genes underwent AS [35,36,37]. Considering the orthologous gene pairs in *Arabidopsis* and rice, 58% of the same AS types were found, suggesting a role for the AS event as an evolutionarily conserved mechanism of post-transcriptional regulation [38]. In this study, five *GS3* splicing variants were found to exist in both diploid and hexaploid Triticeae species (Figure 2C), indicative of the functional significance of ancestral candidate AS. The *GS3.1*, *GS3.2,* and *GS3.3* variants occur in *B. distachyon* and *GS3.1* and *GS3.5* in barley (Figure S9 and Table S3), suggesting that AS of *GS3* is conserved in wheat, barley, and *B. distachyon*. The constitutive variant *GS3.1* is a major variant in both diploid ancestor grasses and hexaploid wheat, portraying a similar situation to most AS events in *Arabidopsis* and cereal crops, in that only one major transcript exists despite the presence of multiple splicing variants [14,39]. The frequency of AS occurrence in wheat tends to decrease after polyploidization, as determined by high-throughput transcriptome sequencing [24]. *GS3* AS occurrence tends to decrease along with the increasing degree of wheat polyploidization, since a higher frequency of *GS3* splicing variants is found in diploid wheat progenitor species compared with hexaploid wheat (Figure 2C).

Polyploidization is often accompanied by changes in genomic structure and gene expression [40]. In hexaploid KN9204 and KN199, a single nucleotide difference causes the absence of one canonical splice junction (AG) in the third intron of *TaGS3*-*7A* (Figure S5), which results in the third intron of *TaGS3*-*7A* differing from that of homeologs *TaGS3-4A* and *TaGS3-7D*. As a consequence, *TaGS3.2* could not be isolated from *TaGS3-7A* in KN9204 and KN199. In our previous reports, *TaGS3-4A* and *TaGS3-7D* exhibited increased transcription abundance compared with *TaGS3-7A* in developing grains, suggesting that *TaGS3-4A* and *TaGS3-7D* play more important roles in grain development [11]. Whether the presence of *TaGS3.2* contributes to increased *TaGS3* expression levels needs further investigation. Our findings here indicate that genomic-structure-triggered AS could contribute to functional diversity among homeologs in bread wheat, which is different from those caused by epigenetic modifications [41,42,43]. Grain size is a complex trait, and different levels of gene function regulation may dominate grain development. Therefore, we inferred the functional significance of *TaGS3* AS event as an evolutionarily conserved mechanism in the regulation of wheat grain weight and size.

### 3.2. Differential Functions and Mechanisms of TaGS3 ASs in Wheat

Some splicing variants compete with the constitutive variant and interfere with its function in a dominant negative manner [17,44]. For instance, FLOWERING LOCUS T (FT) gene *FT2* generates two splicing variants in *B*. *distachyon*, *FT2α* and *FT2β*, which play antagonistic roles in regulating the flowering processes [15]. A similar mechanism was identified here. *TaGS3.2*, *TaGS3.3*, and *TaGS3.4* compete with the constitutive *TaGS3.1* variant in binding to WGB1 to form a functional G_βγ_ heterodimer, respectively (Figure 6 and Figure 7), and thereby regulate grain weight and size. However, the overexpression of *TaGS3.2–3.4* that contain an intact OSR domain coding region (Figure 1B) did not obviously affect grain size (Figure 4C–E), which may be attributed to the low proportion of *TaGS3.2–3.4* and their instability (Figure S7). On the other hand, there is abundant steady-state accumulation of *TaGS3.3*-GFP and *TaGS3.4*-GFP in overexpression lines (Figure 4B, Figure S7). We observed ectopic subcellular localization of *TaGS3.3*-GFP and *TaGS3.4*-GFP in the cytoplasm and nucleus (Figure 3B), with possible traces of functional *TaGS3.3* and *TaGS3.4* localized at the plasma membrane, consistent with the lack of obvious changes in the grain-size phenotype of *TaGS3.3* and *TaGS3.4* overexpression lines. Thus, given the stronger affinity of *TaGS3.2–3.4* to WGB1 (Figure 5C), *TaGS3.2–3.4* functions in antagonistically fine-tuning *TaGS3.1* function.

Furthermore, AS of *FLM* (FLOWERING LOCUS M) and *HAB1* (HYPERSENSITIVE tO ABA1) in Arabidopsis exhibited opposite effects by antagonistic splicing variants, respectively [17,44]. In this study, overexpressing *TaGS3.5* significantly increased grain weight and size, which was opposite to the negative effect of *TaGS3.1* (Figure 4). In the *TaGS3.5* transgenic lines, *TaGS3.5* expression level was markedly increased, while *TaGS3.1* expression level was nearly unchanged (Figure S10), indicating that the positive effect of *TaGS3.5* in enlarging grain size is independent of the *TaGS3.1* expression level. *TaGS3.5* is unable to interact with WGB1 to form active G_βγ_ heterodimer (Figure 6), indicating that the opposing functions of *TaGS3.5* and *TaGS3.1* do not arise from competition. *TaGS3.5* was observed in trace amounts compared to *TaGS3.1* (Figure 2C) but appeared stable in vivo (Figure S7). Thus, it is reasonable that the constant retention of *TaGS3.5* in overexpression lines may obstruct the normal G-protein-mediated signal transduction pathway and lead to the production of large grains.

### 3.3. Functional Diversification of GS3 in Rice and Wheat

*OsGS3* overexpression in rice generated shorter plants with smaller grains and variable grain number [45]. *TaGS3.1* and *TaGS3.5* overexpression here had contrasting and overwhelming effects on morphological grain traits but did not significantly impact plant height and spike characteristics (Figure 4), indicating the functional specialization of *TaGS3* in grain size regulation in wheat. Furthermore, the increased TGW resulting from *TaGS3.5* overexpression consistently contributed to increased individual grain yield (Table S2), indicating the great value of *TaGS3.5* in the genetic improvement of wheat aimed at elevating grain yield.

Nucleotide differences among rice varieties mainly occur in exon regions of *OsGS3*, which regulate grain traits by producing frameshift mutations that cause premature transcriptional termination [10]. Compared with *OsGS3*, sequence polymorphism in *TaGS3* mainly occurs in intron regions [11], which results in AS rather than allelic variations in *TaGS3*. Both the truncated OsGS3 and *TaGS3* function in the determination of grain size, with the truncated *GS3* proteins generated in different ways, namely by allelic variations in rice but by AS in wheat. A nonsense mutation (C to A) in the second exon of *OsGS3* in the Minghui 63 allele results in a PTC and, consequently, truncated OsGS3 devoid of 17 amino acid residues of the OSR domain and the entire C-terminal Cys-rich region, with the production of longer grains [9]. Overexpressing *TaGS3.5* in wheat also leads to longer grains (Figure 4). Notably, *TaGS3.5*, which arises from skipping of the third exon, results in a truncated *TaGS3.5* lacking 15 amino acid residues that include the DPFITI motif that is part of the OSR domain (Figure 1). These observations in both rice and wheat demonstrate that an intact OSR domain is necessary for *GS3* to function as a negative regulator of grain size. The role of *TaGS3.5* with the whole C-terminal Cys-rich region, which is deleted in the truncated OsGS3 in Minghui 63, illustrates the causal relationship between the deletion of the 15 amino acid residues in the OSR domain and the positive regulation of grain size, showing a new hierarchical regulation mechanism mediated by AS variation in wheat.

The C-terminal Cys-rich region (tail) is characteristic of the noncanonical Gγ subunits unique to rice. Variations in the length of the C-terminal tail of G_γ_ protein distinctly affect the final grain phenotype, in which the long-tailed G_γ_ protein gives long grains in rice while the short-tailed and tailless G_γ_ proteins give short grains [45]. Compared with the wild-type *OsGS3*, a 1 bp deletion carried by the Chuan 7 allele results in a truncated OsGS3 that retains the OSR and TM domains but with deletion of most sections of the C-terminal tail, resulting in the production of super short grains [10]. Contrary to this, overexpressing the long-tailed *TaGS3.1* in wheat results in the production of short grains, but overexpressing the tailless *TaGS3.2–3.4* results in medium-sized grains (Figure 4C–F), suggesting that the length of the C-terminal Cys-rich region in *TaGS3* is negatively correlated with grain size in wheat. These observations in both rice and wheat indicate that the C-terminal Cys-rich region of OsGS3 and *TaGS3* have divergent functions in regulating grain weight and size.

## 4. Materials and Methods

### 4.1. Plant Materials

The developing grains of diploid wheat progenitor species TMB02 (*T. boeoticum*, AA), TH02 (*Ae. sharonensis*, SS) and Y199 (*Ae. tauschii*, DD) and *H. vulgare* cv. Morex, *B*. *distachyon* Bd21 and *T*. *aestivum* cv. Kenong 9204 (KN9204, Center for Agricultural Resources Research, Institute of Genetics and Developmental Biology, Chinese Academy of Sciences, Shijiazhuang, China) and Kenong 199 (KN199, Center for Agricultural Resources Research, Institute of Genetics and Developmental Biology, Chinese Academy of Sciences, Shijiazhuang, China) were used to analyze *GS3* sequences as previously described [11].

### 4.2. Isolation of GS3 Splicing Variants

Three sets of primers from 5′ UTR (untranslated region) and 3′ UTR were used to amplify *TaGS3* coding sequences from chromosomes 7A, 4A, and 7D. PCR products were separated by electrophoresis in agarose gels, and the target bands were purified and cloned into the pTOPO-Blunt Simple Vector (Genstar, Beijing, China), which was transformed into DH5α using the heat-shock method (Transgen, Beijing, China).

One set of specific primers corresponding to the CDS (coding sequence) of *TaGS3* was used to amplify the specific *TaGS3* target DNA, the single colonies of which were identified by PCR to distinguish the corresponding sequences of *TaGS3.1* (253 bp), *TaGS3.2* (260 bp), *TaGS3.3* (267 bp), *TaGS3.4* (335 bp), and *TaGS3.5* (208 bp). Positive clones were confirmed by sequencing. Each *TaGS3* splicing variant was analyzed by alignments with the corresponding coding sequence. The number of positive clones in TMB02, TH02, and Y199 was 609, 484, and 932, respectively. In KN9204, the number of positive clones from chromosomes 7A, 4A, and 7D was 858, 803, and 932, respectively. In KN199, the number of positive clones from chromosomes 7A, 4A, and 7D was 787, 820, and 794, respectively.

The coding regions of *HvGS3* and *BdGS3* were cloned from *H. vulgare* cv. Morex and *B*. *distachyon* Bd21, respectively, on the basis of the corresponding reference sequences of *H. vulgare* cv. Lasa Goumang (SDOW01000566) and *B. distachyon* Beauv (XM_014896470.2), the single colonies of which were identified by PCR. Positive clones were confirmed by sequencing. Each *HvGS3* and *BdGS3* splicing variant was analyzed by alignments with the corresponding coding sequence. The number of positive clones in *HvGS3* and *BdGS3* was 47 and 4, respectively. All the primers are listed in Table S4.

The coding sequences of *TaGS3.1*, *TaGS3.2*, *TaGS3.3*, *TaGS3.4*, and *TaGS3.5* from chromosome 4A of KN9204 were subcloned into pJIT163, for expression driven by the ubiquitin promoter, to generate constructs of *TaGS3.1* *OE*, *TaGS3.2* *OE*, *TaGS3.3* *OE*, *TaGS3.4* *OE*, and *TaGS3.5* *OE*, respectively. The constructs were transformed into immature embryos of *T. aestivum* cv. KN199 by gene gun methods [46]. At least 20 independent T_0_ transgenic plants of each construct were identified by PCR.

### 4.3. Phenotype Assessment

Five to seven T_3_ homozygous overexpression lines of each *TaGS3* splicing variant and WT were grown in the field at Luancheng Agro-Ecosystem Experimental Station, CAS, China. A random block design with three replications was performed in which each overexpression line was planted in a 2-row plot that was 2 m long with 25 cm row spacing and 40 seeds per row. Prior to harvest, 10 random plants in the central region of each plot in the main tiller were examined to determine their plant height (PH), spike length (SL), and spike number per plant (SN), and 30 spikes were randomly sampled and examined for determination of the total spikelets per spike (TS), sterile spikelets per spike (SS), and grain number per spike (GN). After harvest, the individual grain yield (GY) of 10 representative plants was recorded, and grains were collected for phenotype assessment. Grain agricultural traits, including grain length (GL), grain width (GW), and thousand-grain weight (TGW), were measured using an SC-G multifunctional seed analyzer (Wanshen, Hangzhou, China).

### 4.4. Plasmid Construct

The coding regions of *WGA1* (G_α_) and *WGB1* (G_β_) were cloned from KN9204 on the basis of the corresponding reference sequences of *WGA1* (G_α_) (MG748862.1) and *WGB1* (G_β_) (XM_037566621) in Chinese Spring (CS). For subcellular localization examination, the coding sequences of five *TaGS3* splicing variants were cloned into the pCAMBIA 1300-35S-GFP vector. In the protein-protein interaction experiments, the coding sequences of the five *TaGS3* splicing variants and DNA sequences to code the full-length *GS3*^1–170^ and the truncated *GS3*^1–66^, *GS3*^1–60^, *GS3*^1–51^, and *GS3*^67–170^ were cloned into pGADT7; then, the coding sequence of *WGB1* was cloned into the pGBKT7 vector for the yeast two-hybrid assay, and the coding sequences of the five *TaGS3* splicing variants and *WGB1* were cloned into pMAL-C2X and pGEX-4T-1, respectively, for the pull-down assay. For the coimmunoprecipitation assay, the coding sequences of the five *TaGS3* splicing variants and *WGB1* were cloned into the pCAMBIA1300-35S-GFP and pCAMBIA1300-35S-FLAG vectors, respectively. For the yeast three-hybrid assay, the coding sequences of *WGA1* were cloned into pGADT7, and the coding sequences of *WGB1* and five *TaGS3* splicing variants were cloned into the pBridge vector. All primers for vector construction are listed in Table S4.

### 4.5. RNA Extraction and qRT-PCR

For tissue-specific expression analysis, different leaf tissues from the plant at the seedling, jointing, booting, and heading stages, along with grain samples of 7, 14, 21, and 28 days post-anthesis were collected from the overexpression lines of the five *TaGS3* splicing variants grown in the field. Total RNA was isolated using an RNA extraction kit (Tiangen, Beijing, China) and quantified by Nanodrop (Thermo, Waltham, MA, USA). First-strand cDNA was synthesized from DNaseI-treated total RNA using the PrimeScript RT Reagent Kit (TaKaRa, Tykyo, Japan) according to the manufacturer’s instructions.

qRT-PCR was carried out in a total volume of 20 μL using the SYBR PCR kit (TaKaRa, Tykyo, Japan) and on a CFX96 Real-Time PCR Detection System (Bio-Rad, Hercules, CA, USA) according to the manufacturer’s instructions. At least three biological replicates were assayed, for which three technical replications were conducted. The *GAPDH* locus served as a normalization control to determine the relative expression level of each splicing variant. Specific primer sequences are listed in Table S4.

### 4.6. Subcellular Localization Assay

The *35S::TaGS3.1-GFP*, *35S::TaGS3.2-GFP*, *35S::TaGS3.3-GFP*, *35S::TaGS3.4-GFP*, and *35S::TaGS3.5-GFP* constructs were introduced into *Agrobacterium tumefaciens* strain GV3101-pMP90, which was then transformed into epidermal cells of *N. benthamiana*. The empty *35S::GFP* vector was used as the control. The transformed *N. benthamiana* leaves were cultivated for 48–72 h before sampling. Confocal imaging was conducted using laser scanning microscopy (Zeiss, Jena, Germany) according to the manufacturer′s instructions.

### 4.7. Yeast Two- and Three-Hybrid Assays

The yeast two-hybrid experiment was performed using the MATCHMAKER GAL4 Two-Hybrid System (Clontech, Mountain View, CA, USA). Briefly, respective prey and bait vectors were cotransformed into yeast strain AH109 using the lithium acetate transformation method. The transformed yeast cells were selected on SD/-Leu-Trp (SD/-LT) dropout medium. Interactions were verified on the SD/-Leu-His-Trp (SD/-LHT) dropout medium with different concentrations of 3-aminotriazole (0 and 2 mM/L).

For the yeast three-hybrid assay, prey vector pGADT7-WGA1 was co-transformed with different pBridge (Clontech, Mountain View, CA, USA) bait vectors *TaGS3.1*-WGB1, *TaGS3.2*-WGB1, *TaGS3.3*-WGB1, *TaGS3.4*-WGB1, and *TaGS3.5*-WGB1 into yeast strain AH109; prey vectors pGADT7-*TaGS3.1*, pGADT7-*TaGS3.2*, pGADT7-*TaGS3.3*, pGADT7-*TaGS3.4*, and pGADT7-*TaGS3.5* were co-transformed with pBridge (Clontech, Mountain View, CA, USA) bait vector WGB1-*TaGS3.1* into yeast strain AH109. A single clone of AH109 from SD/-Met plate was grown on SD/-Leu-Met-Trp (SD/-LMT) medium, and interactions were verified on SD/-Leu-Met-Trp-His (SD/-LMTH) plates with 10 and 20 mM/L 3-aminotriazole.

For liquid β-galactosidase assay with ONPG as substrate, liquid cultures in SD/-LHT with three yeast colonies were inoculated overnight. The calorimetric β-galactosidase assay using the supernatant and the following activity calculation were performed as described in the Clontech Yeast Protocols Handbook.

### 4.8. Pull-Down Assay

*TaGS3.1*-GST, *TaGS3.2*-GST, *TaGS3.3*-GST, *TaGS3.4*-GST, *TaGS3.5*-GST, and WGB1-MBP plasmids were transformed into BL21 cells. Protein purification was performed using Glutathione Sepharose^TM^ 4B (GE Healthcare, Uppsala, Sweden) and Amylose Resin (NEB, Beverly, MA, USA) according to manufacturer instructions. GST beads were washed with GST binding buffer four times to remove the ethanol. Purified GST and *TaGS3.1*-GST, *TaGS3.2*-GST, *TaGS3.3*-GST, *TaGS3.4*-GST, and *TaGS3.5*-GST proteins were incubated with the same volume of GST beads in binding buffer (20 mM Tris-HCl (pH 7.5), 150 mM NaCl, 1% Nonidet P40, with 1 mM phenylmethylsulfonyl fluoride (PMSF) and 0.5 mM dithiothreitol) for 2 h at room temperature. These proteins were centrifuged at 800× *g* for 3 min, and pellets were extensively washed with binding buffer four times to remove redundant proteins. Each purified protein was incubated with WGB1-MBP in binding buffer for another 2 h at room temperature or 4 °C overnight. Next, the mixture was washed four times by binding buffer to remove redundant WGB1-MBP. Samples were then collected and boiled for 5 min in 2× SDS protein loading buffer prior to Western blotting assay. The anti-MBP (TDY Biotech, Beijing, China, 1:5000) and anti-GST antibodies (TDY Biotech, Beijing, China, 1:5000) were used to detect MBP and GST tagged proteins, respectively.

### 4.9. Coimmunoprecipitation Assay

Leaves of 4- to 6-week-old *N. benthamiana* plants were agroinfiltrated with *TaGS3.1*-GFP, *TaGS3.2*-GFP, *TaGS3.3*-GFP, *TaGS3.4*-GFP, *TaGS3.5*-GFP, and WGB1-FLAG when GFP served as control. After 2-day agroinfiltration, leaves were collected and ground to a fine powder in liquid nitrogen using a mortar and pestle. Total protein was then extracted with IP buffer (50 mM Na_2_HPO_4_/NaH_2_PO_4_ (pH 7.4), 150 mM NaCl, 1% Triton X-100, 15% glycerol, 1 mM phenylmethylsulfonyl fluoride (PMSF), and protease inhibitor cocktail (Roche)) and centrifuged at 14,000× *g* at 4 °C three times. Anti-FLAG M2 Affinity Gel (Sigma, St.louis, Mo, USA) was washed four times with PBS buffer and incubated with the extracted proteins at 4 °C for 3 h with gentle shaking. After centrifugation at 600× *g* and 4 °C for 1 min, the precipitated samples were washed five times with washing buffer (50 mM Na_2_HPO_4_/NaH_2_PO_4_ (pH 7.4), 150 mM NaCl, 0.1% Triton X-100, 10% glycerol, 1 mM PMSF, and protease inhibitor cocktail (Roche)). The precipitate was suspended and boiled for 5 min in 2 × SDS protein loading buffer, followed by SDS-PAGE electrophoresis for Western blotting. The anti-GFP (Abcam, Cambridge, UK, 1:2000) or anti-FLAG (Abcam, Cambridge, UK, 1:1000) antibody was used to examine the candidate protein.

### 4.10. Protein Extraction and Immunoblotting Assay

Total protein of 3-day-old seedlings was extracted using the extraction buffer (50 mM Tris, pH 7.5, 150 mM NaCl, 0.1% Triton X-100, 0.2% Nonidet P-40, protease inhibitor cocktail (Roche)). For the immunoblotting assay, the 3-day-old seedlings of *TaGS3.1*-GFP, *TaGS3.2*-GFP, *TaGS3.3*-GFP, *TaGS3.4*-GFP, and *TaGS3.5*-GFP transgenic plants were treated with proteasome inhibitor MG132 (50 μM) and protein synthesis inhibitor CHX (30 μM) for 0, 6, 12, and 20 h, respectively. The wheat shoot (1 g) was lysed in 800 μL extraction buffer before centrifugation at 13,500× *g* for collection of the supernatant, which was boiled for 5 min in 2 × SDS protein loading buffer for Western blotting. Each immunoblot was incubated with primary antibodies anti-GFP (Abcam, Cambridge, UK, 1:2000) and anti-ACTIN (Abcam, Cambridge, UK, 1:5000) for 1 h at room temperature or overnight at 4 °C. Immunoblots were developed using peroxidase conjugated secondary antibody at antirabbit antibody (Abcam, Cambridge, UK, 1:2000) coupled to a chemiluminescence detection system. The obtained Western blot bands were quantified into their relative grey values using the ImageJ software (https://imagej.nih.gov/ij/ accessed on 20 August 2021).

### 4.11. Statistical Analyses

Statistical analyses were based on phenotypic data for grain weight and size. One-way ANOVA was performed in the SPSS System for Windows version 20.0 (IBM Corporation, Armonk, NY, USA) to determine phenotypic differences between overexpression lines and WT, and Tukey tests were conducted to determine the significance of differences.

### 4.12. Accession Numbers

Sequence data from this study can be found in the GenBank database (http://www.ncbi.nlm.nih.gov/ accessed on 15 August 2021) under the following accession numbers: *HvGS3*, SDOW01000566; *BdGS3*, XM_014896470; *WGB1* (G_β_) in Chinese Spring (CS), XM_037566621; *WGA1* (G_α_) in Chinese Spring (CS), MG748862.1; HvGS3, KAE8784062.1; SiGS3, XP_004984061-1; SbGS3, XP_002465152.1; ZmGS3, NP_001354253.1; PhGS3, XP_025797819; OsGS3, XP_015630073.1; BdGS3.1, XP_014751956.

## 5. Conclusions

*TaGS3* undergoes AS and produces five splicing variants that show differential functions in the determination of grain weight and size. AS of *GS3* did not emerge during the process of wheat polyploidization, but pre-existed in the ancestor species of *Triticeae* crops. *TaGS3.1* overexpression significantly reduced grain weight and length, *TaGS3.2–3.4* overexpression did not significantly alter grain weight and size compared to wild type, and *TaGS3.5* overexpression significantly increased grain weight and grain length. *TaGS3* isoforms with an intact OSR domain (TaGS3.1–3.4) interact with WGB1 to form G_βγ_ heterodimers that further interact with WGA1 to form inactive G_αβγ_ heterotrimers. Truncated isoforms *TaGS3.2–3.4* lacking the C-terminal Cys-rich region showed enhanced binding affinity to WGB1 and antagonistically compete with *TaGS3.1* to bind WGB1, while *TaGS3.5* with the incomplete OSR domain does not interact with WGB1. Taken together, the results indicate that *TaGS3* differentially regulates grain size via AS, by which the regulation of grain size is fine-tuned and balanced at the post-transcriptional level.

## Supplementary Materials

The following are available online at https://www.mdpi.com/article/10.3390/ijms222111692/s1.
