# Supplementary material for: Alternative Splicing of TaGS3 Differentially Regulates Grain Weight and Size in Bread Wheat
Source: Int J Mol Sci. 2021 Oct 28;22(21):11692. doi: 10.3390/ijms222111692 (PMC8584009; doi:10.3390/ijms222111692)
Supplement: Supplementary file 1 [file ijms-22-11692-s001.zip › ijms-1397906- supplementary.pdf]

Alternative Splicing of *TaGS3* Differentially Regulates Grain Weight and Size in Bread Wheat

Xiaoli Ren[a, f, #], Liya Zhi[a, f, #], Lei Liu[c], Deyuan Meng[a, f], Qiannan Su[a], Aamana Batool[a, f], Jun Ji[a, e], Liqiang Song[a, e], Na Zhang[a, e], Lin Guo[d], Xigang Liu[d], Junming Li[a, d, e, *], Wei Zhang[b, *]

[a] Center for Agricultural Resources Research, Institute of Genetics and Developmental Biology, The Innovative Academy of Seed Design, Chinese Academy of Sciences, Shijiazhuang 050022, Hebei, China
[b] Hebei University of Economics and Business, Shijiazhuang 050061, Hebei, China
[c] School of Life Science, Huaiyin Normal University, Huaian, 223300, Jiangsu, China
[d] Ministry of Education Key Laboratory of Molecular and Cellular Biology, Hebei Collaboration Innovation Center for Cell Signaling, Hebei Key Laboratory of Molecular and Cellular Biology, College of Life Sciences, Hebei Normal University, Shijiazhuang 050024, China
[e] State Key Laboratory of Plant Cell and Chromosome Engineering, Chinese Academy of Sciences, Beijing 100101, China
[f] The College of Life Science, University of Chinese Academy of Sciences, Beijing, 100049, China

* Corresponding authors: Junming Li, E-mail address: ljm@sjziam.ac.cn;
Wei Zhang, E-mail address: edithor@126.com
[#] Xiaoli Ren and Liya Zhi contributed equally to this work.

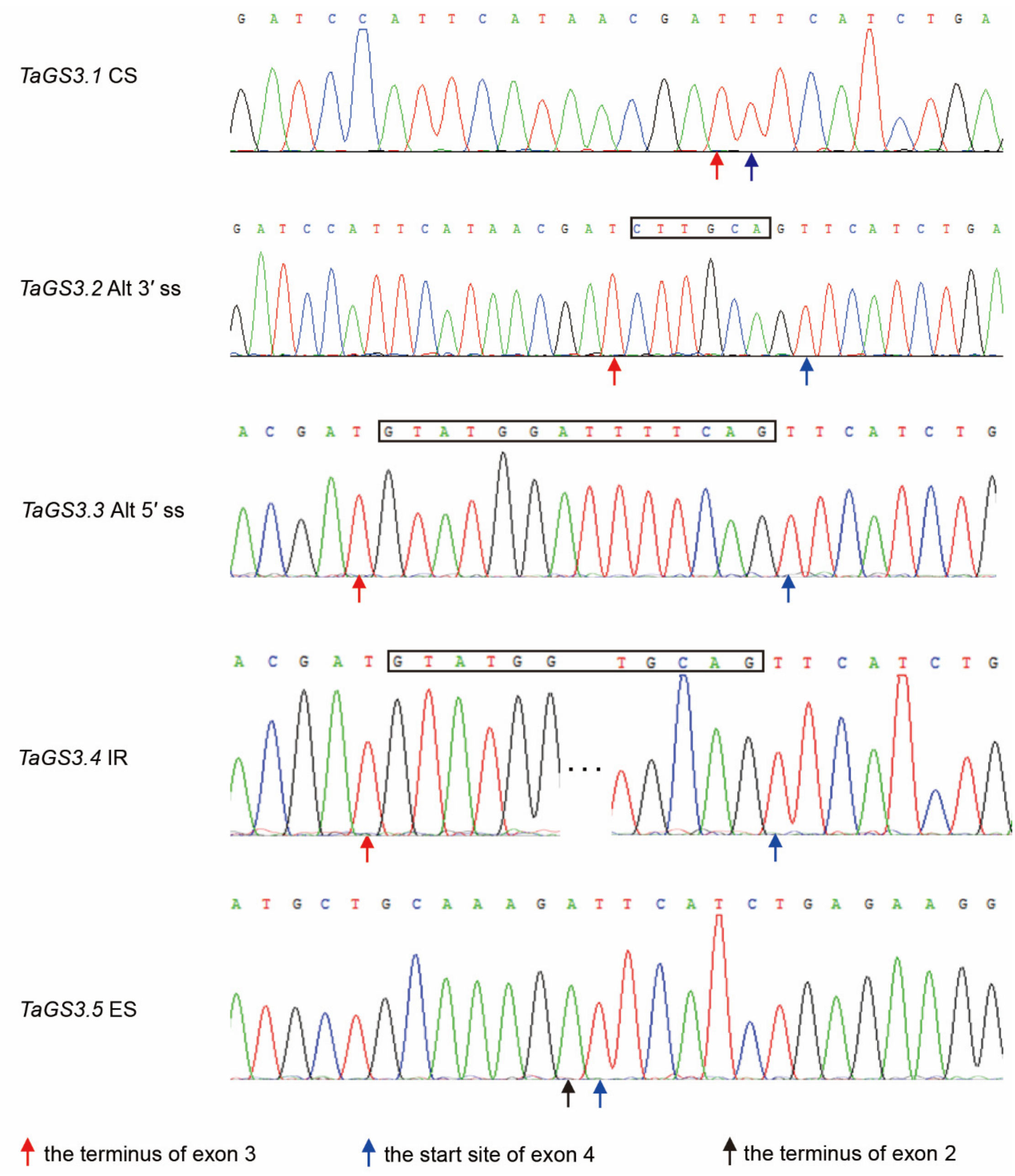

Figure S1. Atlas of Sanger sequencing of *TaGS3* splicing variants.
Black boxes indicate the retention section in intron 3.

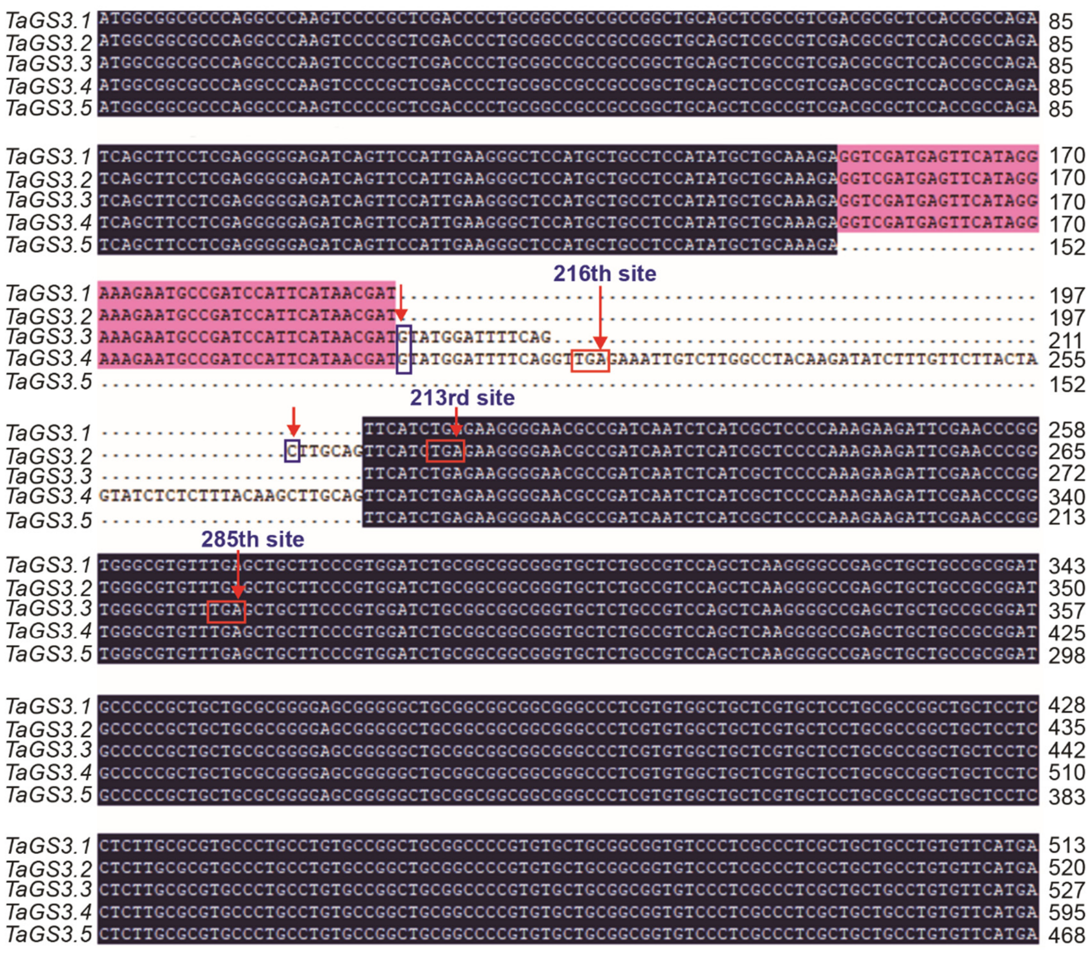

Figure S2. The coding sequence alignment of the *TaGS3* splicing variants.
Blue boxes indicate the sites where *TaGS3.2*, *TaGS3.3* and *TaGS3.4* shift the coding frame, respectively; Red boxes indicate the sites where *TaGS3.2*, *TaGS3.3* and *TaGS3.4* introduce premature termination codons (PTCs) at the 213rd nucleotide site of exon 4, 285th nucleotide site of exon 5 and 216th nucleotide site of exon 4, respectively.

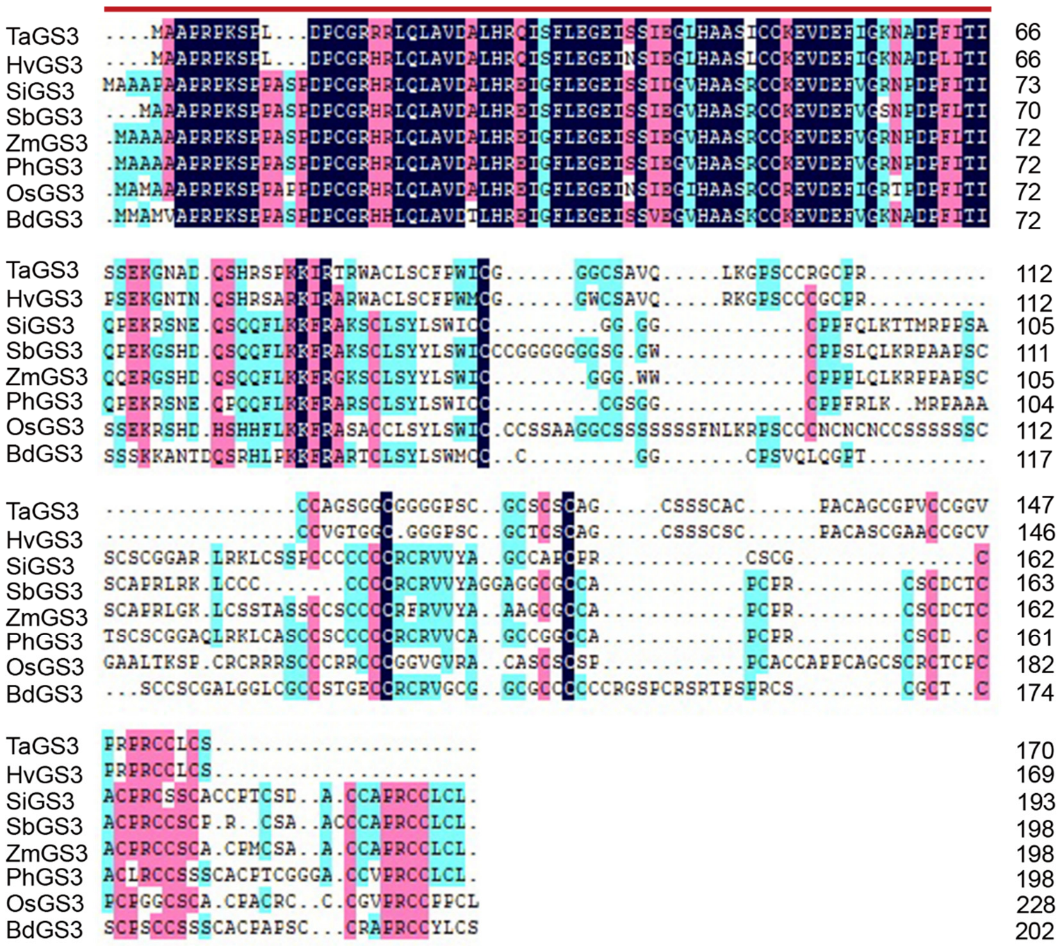

Figure S3. Protein sequence alignment of GS3 in the *Poaceae*.
To identify the homologous proteins of TaGS3 in the *Poaceae*, we conducted a BLAST search with the TaGS3 sequence and found homologs from *H. vulgare* (Hv), *S. italica* (Si), *S. bicolor* (L.) *Moench* (Sb), *Z. mays* (Zm), *P. hallii* (Ph), *O. sativa* (Os) and *B. distachyon* (Bd). Red line indicates the conserved OSR domain.

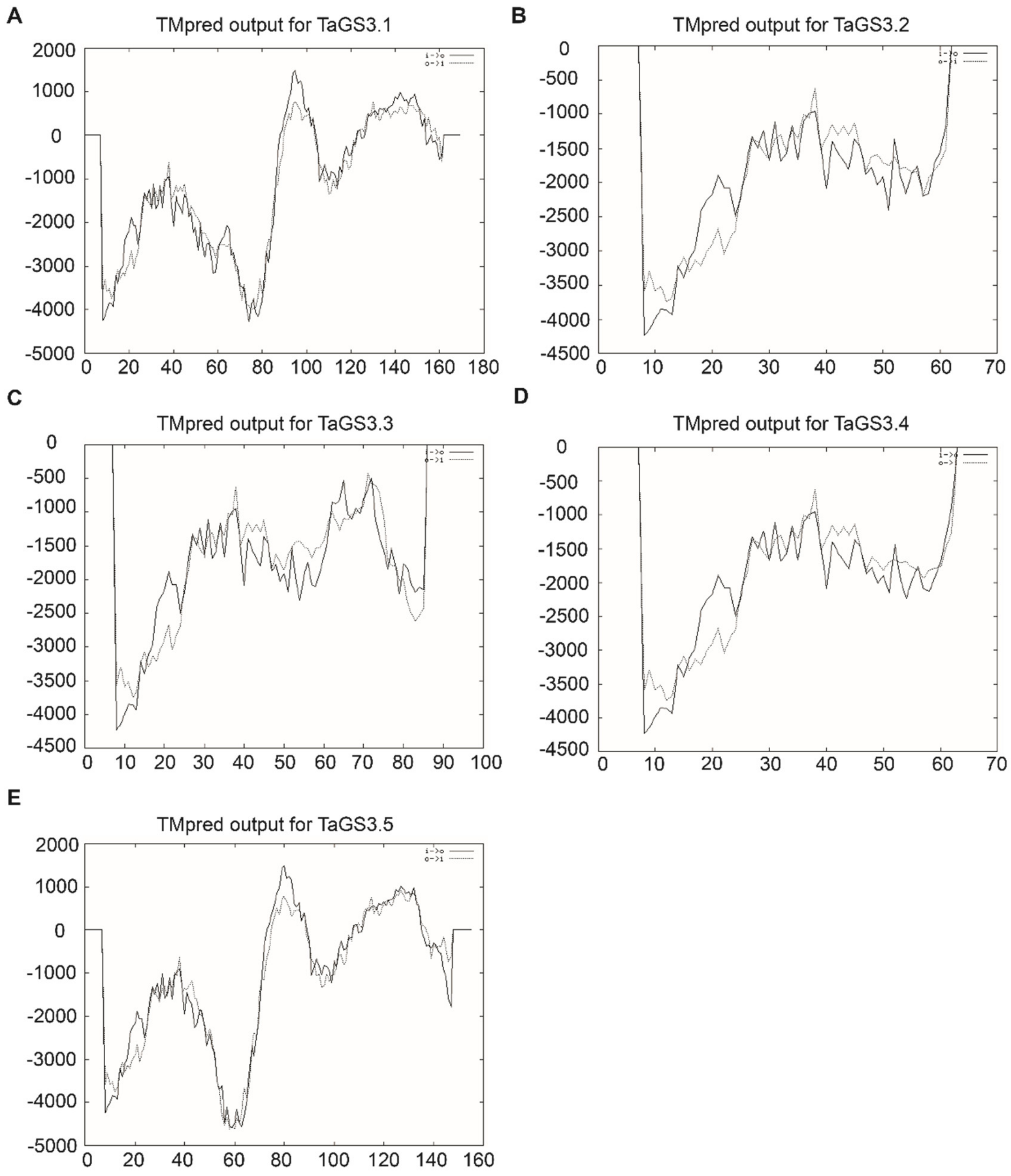

Figure S4. Predicted transmembrane domain of TaGS3 isoforms in bread wheat.
(A) Two predicted transmembrane helices, 87–104 (18 aa) and 127–158 (32 aa), in TaGS3.1.
(B-D) No predicted transmembrane helix in TaGS3.2, TaGS3.3 and TaGS3.4.
(E) Two predicted transmembrane helices, 72–89 (18 aa) and 112–143 (32 aa), in TaGS3.5. i → o, inside → outside; o → i, outside → inside.

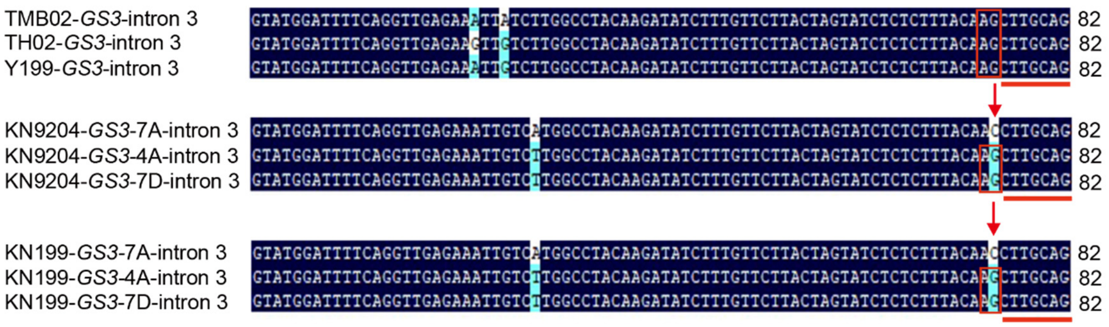

Figure S5. The third intron sequence alignment of *GS3* in diploid wheat progenitors TMB02 (*T. boeoticum*, AA), TH02 (*Ae. sharonensis*, SS) and Y199 (*Ae. Taushii*, DD) as well as *T. aestivum* cv. KN9204 and KN199.

Red lines indicate *GS3.2* harboring seven nucleotides (CTTGCAC) at the terminus of intron 3; Red boxes indicate splicing site AG. Red arrows indicate the nucleotide difference (AG→AC) of *TaGS3-7A* in comparison with *TaGS3-4A* and *TaGS3-7D* in KN9204 and KN199, resulting in absence of one canonical splice site (AG) of *TaGS3-7A*.

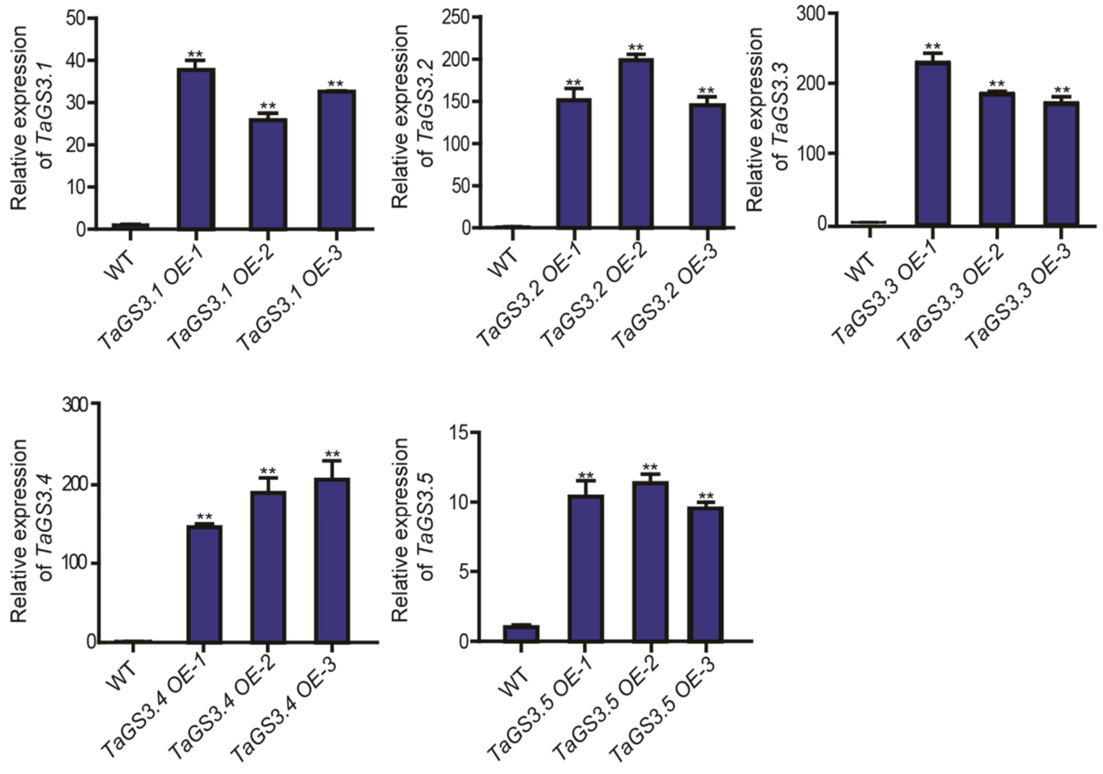

Figure S6. Expression levels of *TaGS3* splicing variants in the seedlings of three representative T_3 overexpression lines (*OE-1* to *OE-3*) and WT.

Gene expression in WT was set to 1. Double asterisk (**) indicates a statistically significant difference (Tukey test, $P < 0.01$). The values are presented as mean ± SEM (n = 3).

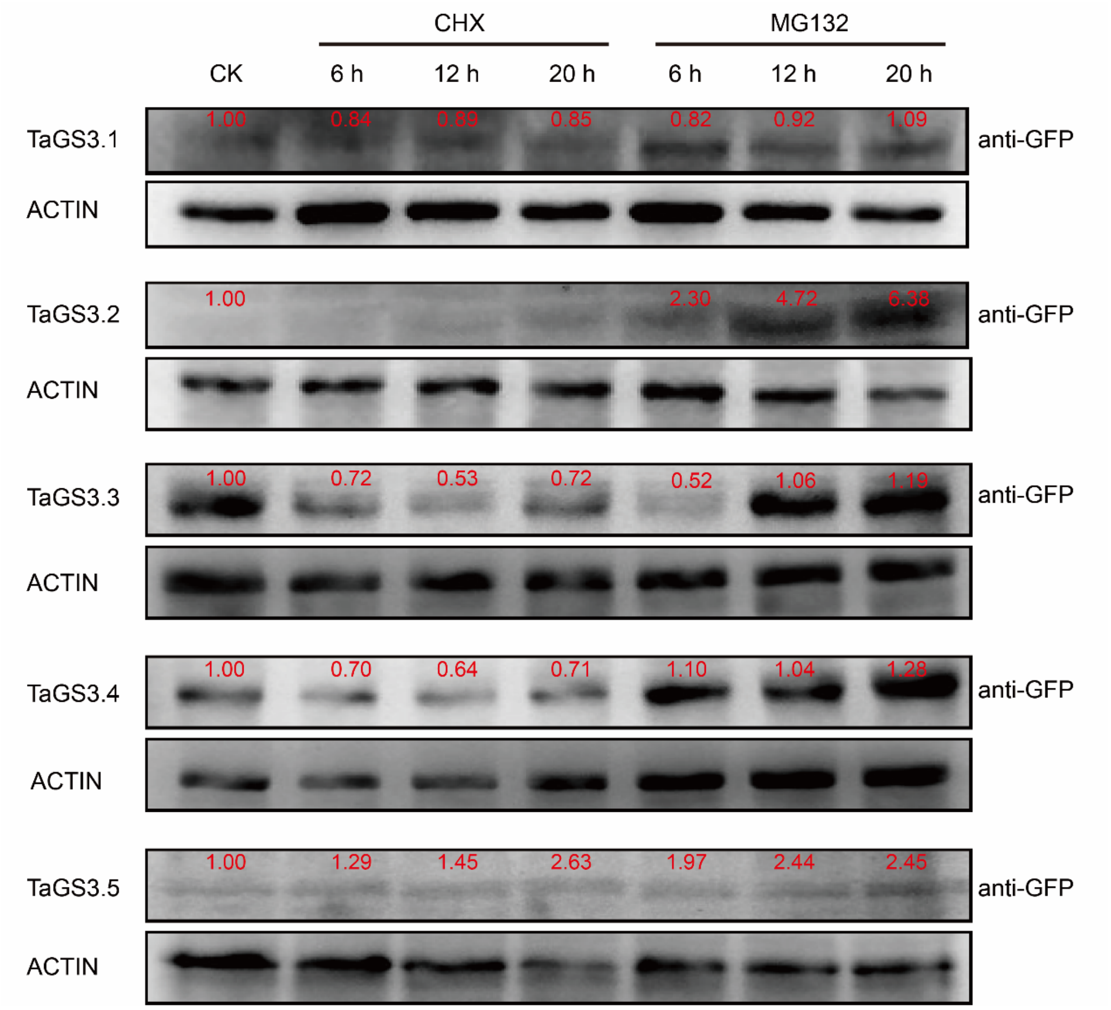

Figure S7. Immunoblot analysis of GFP fused TaGS3 isoforms in the corresponding overexpression lines under 30 μM CHX and 50 μM MG132 treatment, respectively.

Total protein was extracted from the leaves of 3-day-old seedlings for western blotting. CHX, protein synthesis inhibitor (Actidione); MG132, 26S proteasome inhibitor. CK, without treatment; 6 h, 12 h, 20 h indicate hours after treatments. The numbers above each bands indicates the relative grey values.

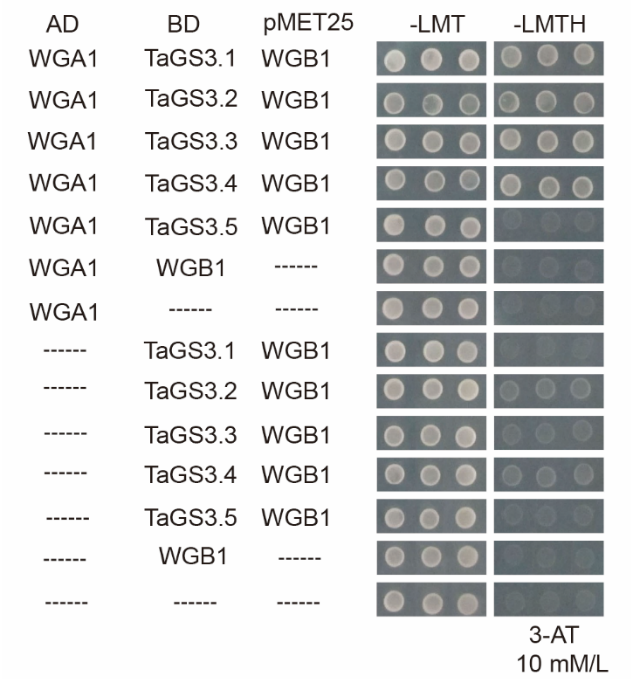

Figure S8. Yeast three-hybrid assay to test interactions of TaGS3 isoforms-WGB1 with WGA1.

The interaction of TaGS3 isoforms-WGB1 with WGA1 was analyzed using fusions with AD (AD-WGA1) and BD (BD-TaGS3 isoforms-WGB1). AD and BD represent empty pGADT7 and pBridge vectors, respectively. The specificity of the stringency of the assay was tested by adding 3-aminotriazole (3-AT, 10 mM/L). Empty vector was used as the negative control.

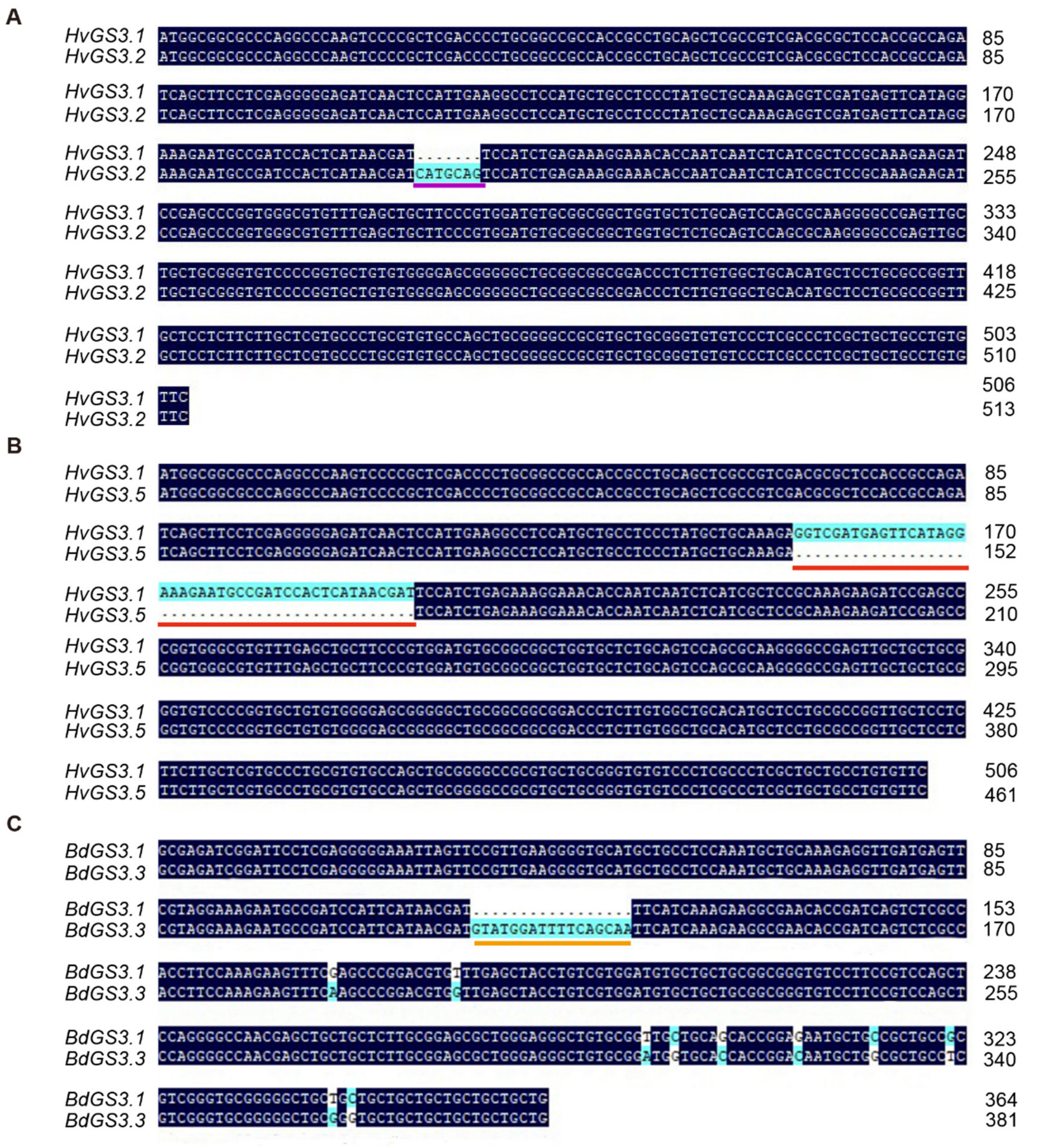

Figure S9. The coding sequence alignment of *GS3* splicing variants in *H. vulgare* and *B. distachyon*.

(A) Sequence alignment of *GS3.1* and *GS3.2* in *H. vulgare*. Purple line indicates the retention of seven nucleotides at the terminus of intron 3.

(B) Sequence alignment of *GS3.1* and *GS3.5* in *H. vulgare*. Red line indicates the third exon (45 bp) skipping.

(C) Sequence alignment of *GS3.1* and *GS3.3* in *B. distachyon*. Orange line indicates the retention of seventeen nucleotide at the start site of intron 3.

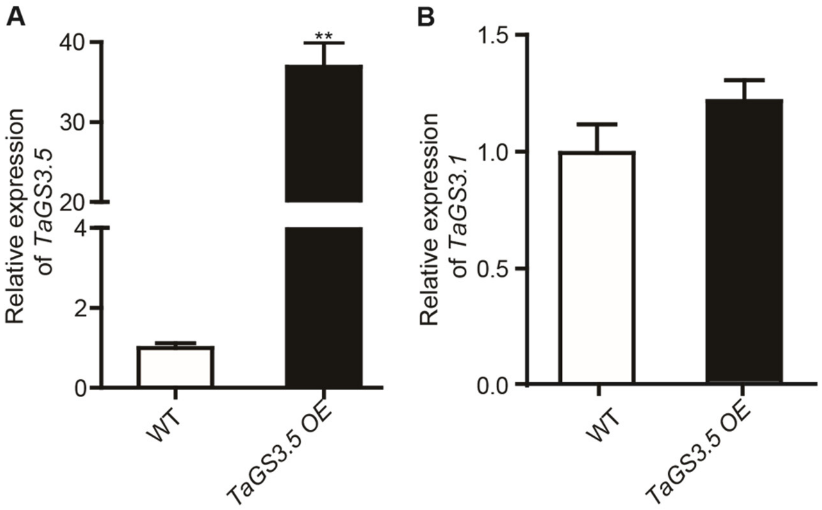

Figure S10. Expression analyses of *TaGS3.1* and *TaGS3.5* in *TaGS3.5* overexpression lines. (A, B) Expression levels of *TaGS3.5* (A) and *TaGS3.1* (B) in 28 DPA grains of WT and *TaGS3.5* overexpression lines. Gene expression in WT was set to 1. Double asterisk (**) indicates a statistically significant difference (Tukey test, $P < 0.01$).

Table S1. Distributions of the five *TaGS3* splicing variants.

AS	Diploid species			KN9204			KN199		
	TMB02	TH02	Y199	7A	4A	7D	7A	4A	7D
GS3.1	92.45%	91.94%	92.27%	98.14%	96.01%	98.11%	98.10%	95.37%	95.59%
GS3.2	1.81%	1.03%	2.36%		3.24%	0.43%		2.20%	0.13%
GS3.3	2.46%	2.27%	2.68%	0.47%	0.50%	0.95%	0.51%	0.49%	1.39%
GS3.4	3.12%	4.75%	2.58%	0.58%	0.12%	0.52%	0.51%	1.95%	2.77%
GS3.5	0.16%		0.11%	0.82%	0.12%		0.89%		0.13%

Table S2. Agronomic attributes of the tested genotypes during three consecutive growing seasons.

GS	Genotypes	PH (cm)	SN	SL (cm)	TS	SS	GN	TGW (g)	GL (mm)	GW (mm)	GY (g/plant)
2018 ~ 2019	KN199	77.57 ± 0.74 a	10.10 ± 0.19 a	8.32 ± 0.08 a	20.25 ± 0.27 a	0.55 ± 0.14a	33.60 ± 0.80 a	47.00 ± 0.16 B	5.96 ± 0.01 B	3.61 ± 0.02 (a) A	13.68 ± 0.02 C
	TaGS3.1 OE	76.35 ± 0.50 a	9.95 ± 0.31 a	8.36 ± 0.09 a	20.50 ± 0.26 a	0.75 ± 0.16 a	33.95 ± 0.53 a	44.23 ± 0.15 C	5.66 ± 0.01 C	3.53 ± 0.02 (b) B	12.89 ± 0.09 B
	TaGS3.2 OE	77.33 ± 0.49 a	9.85 ± 0.20 a	8.33 ± 0.10 a	19.85± 0.30 a	0.85 ± 0.17 a	33.60 ± 0.65 a	46.58 ± 0.22 B	5.92 ± 0.01 B	3.60 ± 0.02 (a) A	13.41 ± 0.07 B
	TaGS3.3 OE	75.88 ± 0.58 a	9.95 ± 0.28 a	8.30 ± 0.07 a	20.55 ± 0.23 a	0.75 ± 0.12 a	34.00 ± 0.87 a	46.62 ± 0.19 B	5.93 ± 0.02 B	3.60 ± 0.02 (a) A	13.55 ± 0.12 B
	TaGS3.4 OE	76.47 ± 0.51 a	10.05 ± 0.20 a	8.31 ± 0.10 a	20.30 ± 0.24 a	0.80 ± 0.19 a	33.70 ± 0.81 a	46.28 ± 0.21 B	5.90 ± 0.01 B	3.59 ± 0.02 (ab) A	13.47 ± 0.06 B
	TaGS3.5 OE	75.98 ± 0.47 a	10.05 ± 0.23 a	8.44 ± 0.10 a	20.20± 0.26 a	0.60 ± 0.18 a	33.90 ± 0.70 a	49.68 ± 0.19 A	6.23 ± 0.03 A	3.66 ± 0.01 (a) A	14.42 ± 0.05 A
2019 ~ 2020	KN199	77.58 ± 0.26 a	10.30 ± 0.18 a	8.81 ± 0.13 a	20.20 ± 0.29 a	1.15 ± 0.17 a	30.55 ± 0.75 a	49.02 ± 0.15 B	6.12 ± 0.01 B	3.63 ± 0.01 (ab) AB	13.02 ± 0.02 B
	TaGS3.1 OE	76.67 ± 0.46 a	10.25 ± 0.30 a	8.61 ± 0.06 a	20.30 ± 0.26 a	1.05 ± 0.17 a	30.70 ± 0.46 a	46.13 ± 0.07 C	5.80 ± 0.02 C	3.49 ± 0.02 (c) C	12.30 ± 0.06 C
	TaGS3.2 OE	77.57 ± 0.53 a	10.20 ± 0.17a	8.68 ± 0.07 a	20.00 ± 0.31a	1.30 ± 0.19 a	30.65 ± 0.64 a	48.64 ± 0.11 B	6.10 ± 0.03 B	3.62 ± 0.01(b) B	13.00 ± 0.04 B
	TaGS3.3 OE	75.81 ± 0.59 a	10.25 ± 0.25 a	8.43 ± 0.15 a	20.25 ± 0.60 a	1.40 ± 0.15 a	30.55± 0.72 a	48.94 ± 0.11 B	6.15 ± 0.01 B	3.63 ± 0.01 (b) AB	13.01 ± 0.03 B
	TaGS3.4 OE	76.40 ± 0.50 a	10.35 ± 0.22 a	8.67 ± 0.05 a	20.40 ± 0.26 a	1.35 ± 0.20 a	30.50 ± 0.76 a	48.77 ± 0.15 B	6.07 ± 0.01 B	3.62 ± 0.01 (b) AB	13.00 ± 0.06 B
	TaGS3.5 OE	75.65 ± 0.54 a	10.25 ± 0.20 a	8.76 ± 0.07 a	20.15 ± 0.20 a	1.35± 0.24 a	30.60 ± 0.50 a	51.37 ± 0.16 A	6.30 ± 0.02 A	3.67 ± 0.01 (a) (AB)	13.67 ± 0.03 A
2020 ~ 2021	KN199	78.50 ± 0.34 a	10.80 ± 0.17 a	8.74 ± 0.11 a	20.30± 0.23a	0.85 ± 0.11 a	33.75± 0.53 a	47.16 ± 0.05 B	5.98 ± 0.01 B	3.61 ± 0.01 A	14.30 ± 0.01 B
	TaGS3.1 OE	77.42 ± 0.74 a	10.75 ± 0.24 a	8.55 ± 0.10 a	20.10 ± 0.26 a	0.85 ± 0.08 a	33.75 ± 0.43 a	44.48 ± 0.07 C	5.67 ± 0.02 C	3.50 ± 0.01 B	13.61 ± 0.07 C
	TaGS3.2 OE	78.16 ± 0.60 a	10.55 ± 0.15 a	8.55 ± 0.09 a	20.20 ± 0.21 a	0.65 ± 0.11 a	33.55 ± 0.48 a	47.29 ± 0.10 B	5.99 ± 0.02 B	3.61 ± 0.01 A	14.20 ± 0.06 B
	TaGS3.3 OE	77.89 ± 0.64 a	10.50 ± 0.14 a	8.47 ± 0.10 a	20.20 ± 0.17 a	0.65 ± 0.13 a	33.65 ± 0.59 a	47.51 ± 0.05 B	6.00 ± 0.02 B	3.62 ± 0.01 A	14.31 ± 0.08 B
	TaGS3.4 OE	77.19 ± 0.53 a	10.65 ± 0.17 a	8.60 ± 0.09 a	20.15 ± 0.18 a	0.65 ± 0.18 a	33.50± 0.66 a	47.21 ± 0.15 B	5.99 ± 0.02 B	3.61 ± 0.01 A	14.14 ± 0.04 B
	TaGS3.5 OE	77.48 ± 0.54 a	10.75 ± 0.16 a	8.67 ± 0.09 a	20.15 ± 0.20 a	0.80 ± 0.16 a	33.85 ± 0.54 a	49.56 ± 0.12 A	6.22 ± 0.02 A	3.64 ± 0.01 A	15.05 ± 0.03 A

Note: GS, growing season; PH, plant height; SN, spike number per plant; SL, spike length in the main tiller; TS, total spikelets per spike; SS, sterile spikelets per spike; GN, grain number per spike; Different lowercases and capital letters indicate significant differences (Tukey test, $P < 0.05$ and $P < 0.01$) among the tested genotypes, respectively. Data are given as mean ± SEM (n = 20).

Table S3. Alternative splicing variants of *TaGS3* in *Poaceae*.

	B. distachyon	*H. vulgare*	*T. boeoticum*	*Ae. sharonensis*	*Ae. Taushii*	*T. aestivum*
GS3.1	√	√	√	√	√	√
GS3.2		√	√	√	√	√
GS3.3	√		√	√	√	√
GS3.4			√	√	√	√
GS3.5		√	√		√	√

Table S4. Primers used in this study.

Primer Name	Primer sequence (5′-3′)
**Primers for amplifying *TaGS3* in diploid species, KN9204 and KN199**	
GS3-7A cDNA 5'UTR-F	CCACCACCGTCCAGCTAGCTAG
GS3-7A cDNA 3'UTR-R	CTGAGCCTGAGACTGGGC
GS3-4A cDNA 5'UTR-F	CGACGACTTCCTGTCTCCTCCTCC
GS3-4A cDNA 3'UTR-R	CAACGCACACAGCAAGAACGAAG
GS3-7D cDNA 5'UTR-F	ACCGGAAACCTTGACCGGC
GS3-7D cDNA 3'UTR-R	CGAGCCTGAGCCTACAG
GS3 CDS-F	ACCGCCAGATCAGCTTCCTC
GS3 CDS-R	CTCGGCCCCTTGAGCTGGACGG
**Primers for amplifying *GS3* in** *B. distachyon*	
Bd21-F1	GCGAGATCGGATTCCTCG
Bd21-R1	CAGCAGCAGCAGCAGCAG
**Primers for amplifying *GS3* in** *H. vulgare*	
HvGS3-F1	ATGGCGGCGCCCAGGCCCAAGTCC
HvGS3-R1	GAACACAGGCAGCAGCGAGGGC
**Primers for overexpression constructs**	
GS3.1OE-F (HindIII)	CCCAAGCTTATGGCGGCGCCCAGGCCC
GS3.1OE-R (BamH Ⅰ)	CGGGATCCTGAACACAGGCAGCAGCGAG
GS3.2OE-R (BamH Ⅰ)	CGGGATCCGATGAACTGCAAGATCGTTATG
GS3.3OE-R (BamH Ⅰ)	CGGGATCCAACACGCCCACCGGGTTC
GS3.4OE-R (BamH Ⅰ)	CGGGATCCACCTGAAAATCCATACATCG
GS3.5OE-R (BamH Ⅰ)	CGGGATCCTGAACACAGGCAGCAGCGAG
**Primers for generating TaGS3 AS transgenic lines**	
GS3.1OE-F (HindIII)	CCCAAGCTTATGGCGGCGCCCAGGCCC
Pjit163-R	GGACACGCTGAACTTGTGG
**Primers for investigating expression patterns of *TaGS3* splicing variants by qRT-PCR**	
GS3-RT-F	GCTCCACCGCCAGATCAGC
GS3.1-RT-R	GTTTCCCTTCTCAGATGAAATC
GS3.2 -RT-F	ACCGCCAGATCAGCTTCCTC
GS3.2-RT-R	TCCCTTCTCAGATGAACTGCAAG
GS3.3-RT-R	GCGTTTCCCTTCTCAGATGAACTGAAAA
GS3.4-RT-R	GGCCATGACAATTTCTCAACC
GS3.5-RT-F	CCTCCATATGCTGCAAAGATTCA
GS3.5-RT-R	AGCATGAGCAGCCACACGAG
**Primers for indentifying *TaGS3* splicing variants in T3 homozygous overexpression lines by qRT-PCR**	
GS3-RT-F	GCTCCACCGCCAGATCAGC
GS3.1-RT-R	GTTTCCCTTCTCAGATGAAATC
GS3.5-RT-F1	ACCGCCAGATCAGCTTCCTC
GS3.5-RT-R1	CTCGGCCCCTTGAGCTGGACGG
GAPDH-QF	TTAGACTTGCGAAGCCAGCA
GAPDH-QR	AAATGCCCTTGAGGTTTCCC
**Primers for Y2H**	
Y2H-GS3.1-F (EcoR Ⅰ)	CCGGAATTCATGGCGGCGCCCAGGCCC
Y2H-GS3.2-R (BamH Ⅰ)	CGGGATCCTCAGATGAACTGCAAGATCGTTATG
Y2H-GS3.3-R (BamH Ⅰ)	CGGGATCCTCAAACACGCCCACCGGGTTC
Y2H-GS3.4-R (BamH Ⅰ)	CGGGATCCTCAACCTGAAAATCCATACATCG
Y2H-GS3.1/GS3.5-R (BamH Ⅰ)	CGGGATCCTCATGAACACAGGCAGCAGCGAG
Y2H-WGB1-F (EcoR Ⅰ)	CGGAATTCATGGCGTCCGTGGCGGAG
Y2H-WGB1-R (BamH Ⅰ)	CGGGATCCTCAGACTATCTTGCGGTGTCC
Y2H-WGA1-F (EcoR Ⅰ)	CGGAATTCATGTCCATGCCCTTGTGTGC
Y2H-WGA1-R (BamH Ⅰ)	CGGGATCCTCACGTCCCCGTTCCTTCC
Y2H-GS3$^{1-170}$-F (EcoR1)	CCGGAATTCATGGCGGCGCCCAGGCCC
Y2H-GS3$^{1-66}$-R (BamH1)	CGGGATCCTCAAATCGTTATGAATGGATCG
Y2H-GS$^{1-60}$-R (BamH1)	CGGGATCCTCAGGCATTCTTTCCTATGAAC
Y2H-GS$^{1-51}$-R (BamH1)	CGGGATCCTCACTCTTTGCAGCATATGGAG
Y2H-GS3$^{67-170}$-F (EcoR1)	CGGAATTCATGTCATCTGAGAAGGGGAAC
Y2H-GS3$^{67-170}$/GS3$^{1-170}$-R (BamH1)	CGGGATCCTCATGAACACAGGCAGCAGC
**Primers for Pull down**	
Pull down-WGB1- F (EcoR Ⅰ)	CCGGAATTCATGGCGTCCGTGGCGGAG
Pull down-WGB1- R (Sal Ⅰ)	ACGCGTCGACTCAGACTATCTTGCGGTGTCC
Pull down-GS3-F (BamH Ⅰ)	CGCGGATCCATGGCGGCGCCCAGGCCC
Pull down-GS3.1-R (EcoR Ⅰ)	CCGGAATTCTCATGAACACAGGCAGCAGCGAG

Pull down-GS3.2- R (EcoR Ⅰ)	CCGGAATTCTCAGATGAACTGCAAGATCG
Pull down-GS3.3-R (EcoR Ⅰ)	CCGGAATTCTCAAACACGCCCACCGGGTTC
Pull down-GS3.4- R (EcoR Ⅰ)	CCGGAATTCTCAACCTGAAAATCCATACATCG
Pull down-GS3.5-R (EcoR Ⅰ)	CCGGAATTCTCATGAACACAGGCAGCAGCGAG
**Primers for Co-IP**	
IP-GS3.1-F (BamH1)	CGGGATCCATGGCGGCGCCCAGGCCC
IP-GS3.1-R (Sma Ⅰ)	TCCCCCGGGTCATGAACACAGGCAGCAGCGAG
IP-GS3.2-R (Sma Ⅰ)	TCCCCCGGGTCAGATGAACTGCAAGATCGTTATG
IP-GS3.3-R (Sma Ⅰ)	TCCCCCGGGAACACGCCCACCGGGTTC
IP-GS3.4-R (Sma Ⅰ)	TCCCCCGGGACCTGAAAATCCATACATCG
IP-GS3.1-F (Xba Ⅰ)	GCTCTAGAATGGCGGCGCCCAGGCCC
IP-GS3.5-R (BamH Ⅰ)	CGGGATCCTGAACACAGGCAGCAGCGAG
IP-WGB1-F (Xba Ⅰ)	GCTCTAGAATGGCGTCCGTGGCGGAG
IP-WGB1-R (EcoR Ⅰ)	CCGGAATTCGACTATCTTGCGGTGTCCAC
**Primers for Y3H**	
Y3H-MCS1-GS3.1-F (EcoR Ⅰ)	CCGGAATTCATGGCGGCGCCCAGGCCC
Y3H-MCS1-GS3.2-R (BamH Ⅰ)	CGGGATCCTCAGATGAACTGCAAGATCGTTATG
Y3H-MCS1-GS3.3-R (BamH Ⅰ)	CGGGATCCTCAAACACGCCCACCGGGTTC
Y3H-MCS1-GS3.4-R (BamH Ⅰ)	CGGGATCCTCAACCTGAAAATCCATACATCG
Y3H-MCS1-GS3.1/3.5-R (BamH Ⅰ)	CGGGATCCTCATGAACACAGGCAGCAGCGAG
Y3H-MCS1-WGB1-F (EcoR Ⅰ)	CGGAATTCATGGCGTCCGTGGCGGAG
Y3H-MCS1-WGB1-R (BamH Ⅰ)	CGGGATCCTCAGACTATCTTGCGGTGTCC
Y3H-MCS2-WGB1-F (Not Ⅰ)	ATAAGAATGCGGCCGCAATGGCGTCCGTGGCGGAG
Y3H-MCS2-WGB1-R (Not Ⅰ)	ATAAGAATGCGGCCGCTCAGACTATCTTGCGGTGTCC
Y3H-MCS2-GS3.1-F (Bgl II)	GAAGATCTATGGCGGCGCCCAGGCCC
Y3H-MCS2-GS3.1-R (Bgl II)	GAAGATCTTCATGAACACAGGCAGCAGC
Y3H-MCS2-GS3.2-R (Bgl II)	GAAGATCTTCAGATGAACTGCAAGATCGTT
Y3H-MCS2-GS3.3-R (Bgl II)	GAAGATCTTCAAACACGCCCACCGGGTTC
Y3H-MCS2-GS3.4-R (Bgl II)	GAAGATCTTCAACCTGAAAATCCATACATCG
Y3H-MCS2-GS3.5-R (Bgl II)	GAAGATCTTCATGAACACAGGCAGCAGCGAG
pBridgeMCS1-F1S	CATCATCATCGGAAGAGAGTAG
pBridgeMCS1-F2L	GCCTCTAACATTGAGACAGCATAG
pBridgeMCS2-F	CCATCCATACAATGGGCCA
pBridgeMCS2-R	TCGGATAAGAAAGCAACACCTG
